# Enhancing the precision limits of interferometric satellite geodesy missions

**DOI:** 10.1038/s41526-022-00204-9

**Published:** 2022-06-08

**Authors:** Lorcán O. Conlon, Thibault Michel, Giovanni Guccione, Kirk McKenzie, Syed M. Assad, Ping Koy Lam

**Affiliations:** 1 Centre for Quantum Computation and Communication Technology, Department of Quantum Science, Australian National University, Canberra, ACT 2601 Australia; 2 Centre for Gravitational Astrophysics (CGA), Research School of Physics, The Australian National University, Canberra, ACT 2601 Australia; 3 ARC Centre of Excellence for Gravitational Wave Discovery (OzGrav), Research School of Physics, The Australian National University, Canberra, ACT 2601 Australia; 4 School of Physical and Mathematical Sciences, Nanyang Technological University, Singapore, 639673 Republic of Singapore

**Keywords:** Optical physics, Optical techniques, Applied physics

## Abstract

Satellite geodesy uses the measurement of the motion of one or more satellites to infer precise information about the Earth’s gravitational field. In this work, we consider the achievable precision limits on such measurements by examining approximate models for the three main noise sources in the measurement process of the current Gravitational Recovery and Climate Experiment (GRACE) Follow-On mission: laser phase noise, accelerometer noise and quantum noise. We show that, through time-delay interferometry, it is possible to remove the laser phase noise from the measurement, allowing for almost three orders of magnitude improvement in the signal-to-noise ratio. Several differential mass satellite formations are presented which can further enhance the signal-to-noise ratio through the removal of accelerometer noise. Finally, techniques from quantum optics have been studied, and found to have great promise for reducing quantum noise in other alternative mission configurations. We model the spectral noise performance using an intuitive 1D model and verify that our proposals have the potential to greatly enhance the performance of near-future satellite geodesy missions.

## Introduction

The possibility of using a pair of satellites to measure the Earth’s gravitational field was first proposed by Wolff in 1969^1^. Based on this premise the GRACE mission was launched in 2002, providing scientists with the tools necessary to recover the Earth’s gravitational field with unprecedented precision^2–4^. GRACE consisted of two satellites which orbited the Earth on very similar trajectories, with an on-board ranging system which measured the satellite separation to great accuracy. The original GRACE mission used a microwave ranging system^5^, and the second generation mission, GRACE Follow-On (GRACE-FO), included the addition of a laser ranging interferometer (LRI)^6,7^. Even though the LRI was not designed to be the main instrument in GRACE-FO, and was included to demonstrate improved sensitivity for future missions, it provided a promising indication of future precision enhancement^6^. This is the first intersatellite optical interferometer, and also serves as an important technological demonstration for the Laser Interferometer Space Antenna (LISA)^8^, a planned space-borne gravitational wave detector.

The advantage of a satellite-based LRI is not limited to metrological missions. There are many reasons to believe the future of the quantum internet lies in space^9–11^, and GRACE-FO with its LRI represents an important step towards this vision. On this front, there has been much progress towards a space-based quantum key distribution network^12–15^, and it is only a matter of time before satellite-to-satellite links are employed to greatly extend the distance for secure communication. The GRACE-FO mission already demonstrates some crucial elements of continuous variable quantum communications; both relying on coherent laser links over large distances. Thus, the mission is of great importance, even beyond its contribution to our knowledge of the Earth’s gravitational field.

The interferometric measurement used on GRACE-FO works by measuring the relative phase, in cycles, between the lasers on-board each satellite. Such a measurement intrinsically has two fundamental noise sources: laser phase noise^6^, caused by imperfect laser stability, and unavoidable quantum noise^16^ caused by photon number fluctuations. In addition to the LRI, the GRACE-FO mission requires accelerometers on board both satellites to distinguish gravitational (signal) and non-gravitational (noise) accelerations^17^. The non-gravitational accelerations come from a variety of sources, such as aerodynamic drag and solar radiation pressure. It is necessary to remove the non-gravitational accelerations from the measurement in order to get a faithful estimate of the gravitational field, hence non-gravitational accelerations can be thought of as another noise source. This noise can be removed using the accelerometer measurement data at the expense of introducing accelerometer instrument noise. We shall use the term accelerometer noise for any noise associated with the non-gravitational acceleration and its removal, i.e., both accelerometer instrument noise and non-gravitational accelerations. Thus, the total measurement noise comes from the accelerometer noise, as well as the laser phase noise and quantum noise from the interferometric measurement. Although in this paper we only consider measurement noise, there are other noise sources which may limit the gravitational field recovery, such as aliasing noise^18^ and tilt-to-length coupling error^19^. This paper is divided into three analyses, discussing the possibility of diminishing the effects of each of the measurement noise sources in turn. First we show that time delay interferometry (TDI), which has been proposed for LISA^20–24^, is a powerful tool for mitigating the effects of laser phase noise. TDI has been considered before for GRACE-FO, however not to enhance the GRACE-FO mission but as a technological demonstration for LISA^25^. We also show that appropriate formations of different mass satellites can be used to reduce accelerometer noise and laser phase noise simultaneously. Multi-satellite formation flying has been suggested^26–28^, however not as a technique for removing measurement noise but to enhance the gravitational signal. Finally we turn to a quantum-limited GRACE, considering what happens when quantum noise is the dominant noise source of such a mission. In this situation, techniques from quantum optics can reduce the quantum noise and we find a whole new regime for satellite geodesy. Indeed, when quantum noise limited, the optimal satellite separation could shrink from hundreds of kilometres to a few kilometres. This suggests that future gravitational recovery missions, perhaps in other planetary settings, may look very different from today’s GRACE-FO mission.

## Results

Before presenting our main results, we first describe the models we shall use for the gravitational signal and measurement noise.

### Gravitational signal

GRACE-FO measures sub-micrometre changes in the satellite separation through changes in the phase of the laser light travelling between the satellites. The phase change is then converted to a change in the separation, or range, between the two satellites, which is in turn converted to a range acceleration. The measured non-gravitational accelerations, along with other forces, such as tidal gravitational forces^29^ and other non-tidal forces^30^ which contribute to the background gravitational field, are then removed from this range acceleration. The remaining range acceleration of the two satellites is used to estimate the Earth’s local gravitational field.

**Fig. 1:**
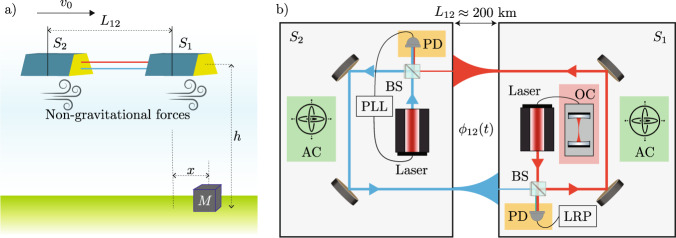
Schematic of satellite geodesy. **a** Current GRACE-FO formation. Two satellites separated by a distance *L*_12_ fly over a mass *M* at a height *h* above the Earth. The differential acceleration allows information about the gravitational field to be recovered. **b** Detailed schematic showing the main noise sources in the current GRACE-FO mission, highlighted in different colours. Satellite *S*_1_ is designated as the master satellite and sends out a laser beam stabilised to an optical cavity (OC), which determines the laser phase noise (red box). The second satellite, *S*_2_, returns a laser beam phase locked to this at a 10 MHz offset. Inherent fluctuations in the number of photons manifest as quantum noise, highlighted at the photodetection stage (orange boxes). There are also non-gravitational forces which affect the motion of the two satellites (highlighted in blue in **a**. Non-gravitational acceleration arises from a variety of sources, including aerodynamic drag and solar radiation pressure. Non-gravitational acceleration can be measured and removed at the expense of introducing accelerometer instrument noise (green box). Measurement instruments include accelerometer (AC), photodetector (PD), beam-splitter (BS), phase locked loop (PLL) and laser ranging processor (LRP).

In reality this is done considering a spherical harmonic expansion of the Earth’s gravitational potential. Instead, we turn to a simpler linear model^31^ to obtain analytic solutions for the motion of a body in such a field. Although we are primarily concerned with the measurement noise, which is largely unaffected by this simplification, this simplified model may fail to capture the full complexity of real-world satellite geodesy and instead provides an indication of what techniques may be beneficial in reality. A schematic of this model is shown in Fig. 1a. We consider two satellites at a height *h* above the ground, separated by a distance *L*_12_. The first satellite is a distance $\sqrt{{h}^{2}+{x}^{2}}$ from a point mass *M* located on the surface of the Earth. Both satellites are initially travelling with velocity *v*_0_. In the frequency domain the measured range acceleration between the two satellites is given by ref. ^31^ as

1 $$| {a}_{{{{\rm{R}}}}}(f)| =\frac{16\pi fG{{{\rm{M}}}}}{{v}_{0}^{2}}\left| {K}_{0}\left(\frac{2\pi f}{{f}_{h}}\right)\right| \left| \,{{\mbox{sin}}}\,\left(\frac{2\pi f}{{f}_{L}}\right)\right|,$$

where *G* = 6.67 × 10^−11^ m^3^ kg^−1^ s^−2^ is the gravitational constant, *f*_*h*_ = *v*_0_/*h*, *f*_*L*_ = 2*v*_0_/*L*_12_ and *K*_0_ is the zeroth order modified Bessel function of the second kind (see Supplementary Note 1 for a full derivation). This is purely range acceleration. For parameters relevant to the current GRACE-FO mission (*h* ≈ 500 km and *L*_12_ ≈ 200 km) this signal is approximately linear in *L*_12_ in the low frequency limit.

### Measurement noise

The measurement noise in this simple model comes in three forms, with two possible sources of accelerometer noise. Figure 1 shows a detailed schematic of the current GRACE-FO mission with the main noise sources highlighted in different colours. Non gravitational forces acting on the satellite contribute a non-gravitational phase shift to the laser light (highlighted in blue in Fig. 1a). Thus, the total measured phase shift is *ϕ*(*t*) = *ϕ*^g^(*t*) + *ϕ*^ng^(*t*), where superscript (n)g denotes the phase shift due to (non-)gravitational forces. Before the range acceleration inferred from the measured phase can be compared to the expected acceleration based on the current best known gravitational field, the non-gravitational accelerations are removed using accelerometer data. This adds accelerometer instrument noise to the measurement with root power spectral density of the form (green box in Fig. 1b)

2 $$\sqrt{{S}_{{\rm{AN}}}(f)}=\sqrt{2}{a}_{0}\sqrt{1+{\left(\frac{{f}_{k}}{f}\right)}^{2}},$$

where the maximum sensitivity of the accelerometer is defined by the acceleration white noise *a*_0_ and the low frequency noise of the accelerometer is defined by *f*_*k*_ = 5mHz^32^. The current GRACE-FO mission has ${a}_{0}\approx 100\,{{{\mbox{pm s}}}}^{-2}{\sqrt{{{\mbox{Hz}}}}}^{-1}$ and it is anticipated that the next generation of GRACE will have ${a}_{0}\approx 1\,{{{\mbox{pm s}}}}^{-2}{\sqrt{{{\mbox{Hz}}}}}^{-1}$^31^. Note however, that this is only an approximate model for the accelerometer instrument noise on the GRACE-FO mission.

The LRI measures the phase between the two satellites with sub-micrometer precision, however there is some laser phase noise remaining in this measurement. The current instrument makes two measurements, one on each satellite, which are combined into a single useful measurement. After removing the non-gravitational element the remaining signal is

3 $$2{\hat{\phi }}_{12}^{\,{{\mbox{g}}}}(t)=2{\phi }_{12}^{{{\mbox{g}}}\,}(t)+{C}_{1}(t-2{\tau }_{12})-{C}_{1}(t)+{N}_{12}(t)\,,$$

where ${\hat{\phi }}_{ij}^{\,{{\mbox{g}}}\,}$ denotes the estimate of the gravitational phase shift measured at satellite *i* using the light arriving from satellite *j*, *τ*_*ij*_ is the single-trip time of flight for light along that arm, *C*_*i*_(*t*) denotes the phase noise of the laser at satellite *i* at time *t* and *N*_*ij*_(*t*) denotes other noise sources in the measurement of light arriving at satellite *i* from satellite *j* (i.e., accelerometer instrument noise and quantum noise). For small *τ*_12_ (*L*_12_/*c* ≪ 1), this implies that the laser phase noise is proportional to satellite separation, as discussed in Supplementary Note 2. For increased laser stability, one of the lasers is locked to an optical cavity and the second laser is then locked to the first. The current GRACE-FO mission requirement on the laser phase noise (red box in Fig. 1b) has the following form

4 $$\begin{array}{l}\sqrt{{S}_{{{\mbox{LPN}}}}(f)} \,<\,{x}_{c}\sqrt{1+{\left(\displaystyle{\frac{3{\,{\mbox{mHz}}}}{f}}\right)}^{2}}\\ \,\,\,\quad\qquad\qquad\times\, \sqrt{1+{\left(\displaystyle{\frac{10{\,{\mbox{mHz}}}}{f}}\right)}^{2}}\left(\displaystyle{\frac{{L}_{12}}{220\,{{\mbox{km}}}}}\right){(2\pi f)}^{2},\end{array}$$

where ${x}_{c}\approx 80\,{{\mbox{nm}}}{\sqrt{{{\mbox{Hz}}}}}^{-1}$^6^ is a constant which we call laser white noise. However, the actual mission performance of the optical cavity exceeded this requirement. The actual laser phase noise performance is at the level of the cavity thermal noise

5 $$\sqrt{{S}_{{{\rm{LPN}}}}(f)}=\frac{{(2\pi f)}^{2}{x}_{T}{L}_{12}}{\sqrt{f}}\,,$$

where *x*_*T*_ ≈ 1 × 10^−15^ is a constant which we call laser thermal noise^31^.

Both satellites in the GRACE-FO mission have a photoreceiver to measure the incoming light and fluctuations in the received photon number manifest as quantum noise. The quantum noise spectrum has the following form (orange box in Fig. 1b)

6 $$\sqrt{{S}_{{{\rm{QN}}}}(f)}=\sqrt{2}{(2\pi f)}^{2}{\delta }_{QN}\,,$$

where *δ*_QN_ is a factor dependent on the amount of received power (discussed in more detail in Supplementary Note 3) and the factor of $\sqrt{2}$ comes from the fact that two measurements are made. A received power of 1 nW corresponds to a quantum noise level of *δ*_QN_ ≈ 1 pm${\sqrt{{{\mbox{Hz}}}}}^{-1}$ (note that this assumes homodyne detection and near-unity detection efficiency). Quantum noise is not presently a limiting factor, but it may be once other sources of noise are addressed and the interferometer becomes quantum-limited. These signal and noise spectra allow a complete characterisation of this model and are summarised in Fig. 6a.

### Time delay interferometry for geodesy

**Fig. 2:**
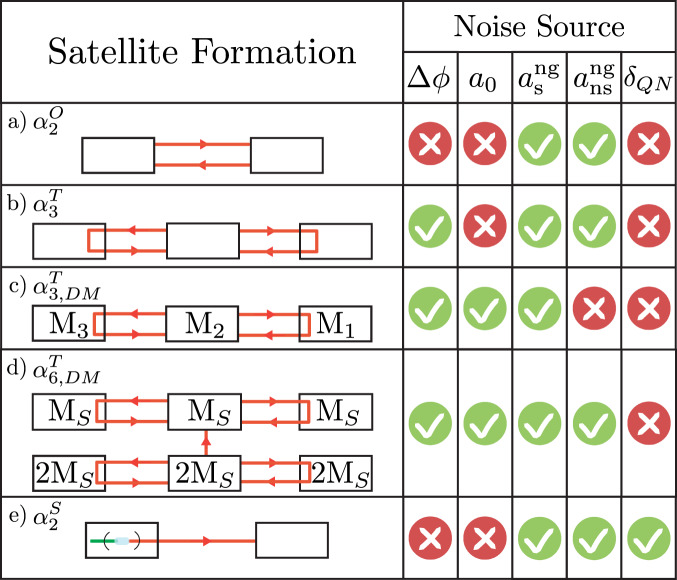
Possible future mission formations. Possible formations are denoted by ${\alpha }_{j,k}^{i}$, where *i* represents the technique being used, *j* represents the number of satellites used and *k* indicates if satellites of different mass are necessary. **a** Original two satellite formation ${\alpha }_{2}^{O}$, **b** three satellite formation with TDI ${\alpha }_{3}^{T}$, **c** three satellite differential mass formation with TDI ${\alpha }_{3,DM}^{T}$, **d** six satellite differential mass formation with TDI ${\alpha }_{6,DM}^{T}$ and **e** two satellite formation using squeezed light ${\alpha }_{2}^{S}$. These allow the removal of various noise sources; laser phase noise Δ*ϕ*, accelerometer instrument noise *a*_0_, stationary and non-stationary non-gravitational accelerations, ${a}_{\,{{\mbox{s}}}}^{{{\mbox{ng}}}\,}$ and ${a}_{\,{{\mbox{ns}}}}^{{{\mbox{ng}}}\,}$, respectively, and quantum noise *δ*_*QN*_. Formations ${\alpha }_{3}^{T}$, ${\alpha }_{3,DM}^{T}$ and ${\alpha }_{6,DM}^{T}$ all use more than two satellites and so can remove laser phase noise through TDI. Formations ${\alpha }_{2}^{O}$, ${\alpha }_{3}^{T}$ and ${\alpha }_{2}^{S}$ use accelerometers and so the ${a}_{\,{{\mbox{s}}}}^{{{\mbox{ng}}}\,}$ and ${a}_{\,{{\mbox{ns}}}}^{{{\mbox{ng}}}\,}$ terms are removed from the measurement at the expense of accelerometer instrument noise. Formations ${\alpha }_{3,DM}^{T}$ and ${\alpha }_{6,DM}^{T}$ do not use accelerometers and the non-gravitational accelerations are removed through appropriate combinations of the measurements, made possible by the different satellite masses. No scheme can completely remove quantum noise as each additional measurement adds a new source of quantum noise, however it can be reduced through the use of squeezed light as shown in formation ${\alpha }_{2}^{S}$.

**Fig. 3:**
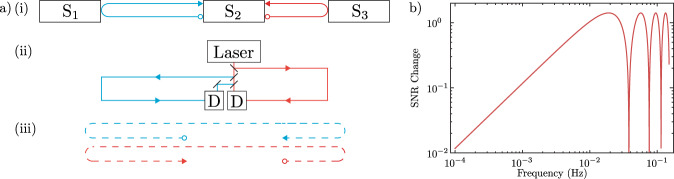
TDI applied to GRACE-like mission. **a** Schematic showing how the different length optical paths are converted to optical paths of the same effective length through TDI. (i) shows the true optical paths between the three satellites. (ii) shows how these optical paths are measured with the same laser. Finally (iii) shows how the effective optical path lengths for the two beams are equal after TDI. D represents the detection process, either homodyne or heterodyne detection. The different colours for the optical paths are for illustrative purposes only. **b** Ratio of the SNR with TDI to the SNR without TDI for both quantum noise and accelerometer noise assuming a satellite velocity of *v*_0_ = 7600 m/s and satellite separation of *L*_12_ = 200 km. There are certain frequencies where the SNR is enhanced.

We now show how TDI can be used to significantly reduce laser phase noise (Eqs. (4), (5)). TDI is a post-processing technique that uses multiple measurements, recombined with different time offsets, to cancel out common-mode noise^20^. To this end we consider multiple satellite formations with several measurements being made. Formation ${\alpha }_{3}^{T}$, from Fig. 2b, with a single laser on the middle satellite is examined in detail, however many other combinations are possible, including combinations with multiple lasers, discussed in Supplementary Note 4. For formation ${\alpha }_{3}^{T}$ the middle satellite acts as the master satellite for the fleet. Light is split into four paths using beamsplitters, with two light beams being sent to the two outer satellites, where they are reflected back to the middle satellite (in practice this would be implemented using phase locked loops and second lasers, as shown in Fig. 1b, rather than mirrors). At the middle satellite two independent measurements are made, using the two light beams which remained on the middle satellite as local oscillators, shown in Fig. 3a. A similar TDI combination has been considered before for detecting gravitational waves^33^. After removing the non-gravitational phase shift from the measurement using accelerometer data at time *t* ($a(t)=\ddot{\phi }(t)\lambda /2\pi$) the measured signal is

7 $$2{\hat{\phi }}_{21}^{\,{{\mbox{g}}}}(t)=2{\phi }_{21}^{{{\mbox{g}}}\,}(t)+{C}_{2}(t-2{\tau }_{21})-{C}_{2}(t)+{N}_{21}(t)\,,$$

8 $$2{\hat{\phi }}_{23}^{\,{{\mbox{g}}}}(t)=2{\phi }_{23}^{{{\mbox{g}}}\,}(t)+{C}_{2}(t-2{\tau }_{23})-{C}_{2}(t)+{N}_{23}(t)\,,$$

using the same notation as before. In order to cancel out the laser phase noise the effective optical path length needs to be the same for both beams, as is illustrated in Fig. 3a. The following combination of the blue (LHS) and red (RHS) optical paths achieves this:

9 $$\begin{array}{ll}{{\mbox{}}}2([{\hat{\phi }}_{21}^{\,{{\mbox{g}}}\,}(t)-{\hat{\phi }}_{23}^{\,{{\mbox{g}}}\,}(t)]-[{\hat{\phi }}_{21}^{\,{{\mbox{g}}}\,}(t-2{\tau }_{23})-{\hat{\phi }}_{23}^{\,{{\mbox{g}}}\,}(t-2{\tau }_{21})])\\ ={{\mbox{}}}2[{\phi }_{21}^{\,{{\mbox{g}}}}(t)-{\phi }_{21}^{{{\mbox{g}}}}(t-2{\tau }_{23})+{\phi }_{23}^{{{\mbox{g}}}}(t-2{\tau }_{21})-{\phi }_{23}^{{{\mbox{g}}}\,}(t)]\\ +\,{{\mbox{}}}({N}_{21}(t)-{N}_{21}(t-2{\tau }_{23}))-({N}_{23}(t)-{N}_{23}(t-2{\tau }_{21}))\,.\end{array}$$

Converting to the frequency domain gives a signal which can be compared to the original scheme where TDI was not employed, ${\alpha }_{2}^{O}$. In order to do so we make the simplification that both satellite separations are initially equal, *L*_12_ = *L*_23_ = *L*. Each ${\phi }_{ij}^{\,{{\mbox{g}}}\,}$ term corresponds to the differential acceleration of one pair of satellites (Eq. (1)). ${\phi }_{23}^{\,{{\mbox{g}}}\,}(t)$ is then equal to ${\phi }_{21}^{\,{{\mbox{g}}}\,}(t)$, delayed by the time-period (*v*_0_/*L*). The TDI signal includes an additional delay on each ${\phi }_{ij}^{\,{{\mbox{g}}}\,}$ term, but by a time-period corresponding to the time of flight of the light (the previous delay corresponded to the time of flight of the satellite). This gives the signal in the frequency domain after TDI, as:

10 $$\begin{array}{ll}\left|{a}_{{{\mbox{R,TDI}}}}(f)\right|=\frac{64\pi fGM}{{v}_{0}^{2}}\left|{K}_{0}\left(\frac{2\pi f}{{f}_{h}}\right)\right|\\ \qquad\qquad\qquad\times \left|\,{{\rm{sin}}}\,{\left(\frac{2\pi f}{{f}_{L}}\right)}^{2}\right|\left|\,{{\rm{sin}}}\,\left(\frac{\pi f}{{f}_{c}}\right)\right|\,,\end{array}$$

where *f*_*c*_ = *c*/2*L*, see Supplementary Note 5 for more detail. Clearly if the distance along the two arms is the same, TDI is not necessary as the measured signals can simply be subtracted with no time delay to remove the laser phase noise. However, in practice all three satellites will fly along slightly different trajectories and experience different non-gravitational accelerations. Therefore, even if the satellites are approximately evenly spaced, TDI will still be necessary to cancel the laser phase noise. In Supplementary Note 6 we consider the signal after using TDI when the two arm lengths (*L*_12_ and *L*_23_) are different, however, this does not significantly affect our results.

### Signal-to-noise ratio after TDI

Although laser phase noise can in principle be completely cancelled, imperfections in our knowledge of the satellite positions will hinder how well the laser phase noise is suppressed. In Supplementary Note 7 we show that the noise spectrum of the laser phase noise after TDI, $\sqrt{{S}_{{{{\rm{LPN}}}}_{{{\rm{left-over}}}}}(f)}$, is approximately given by

11 $$\sqrt{{S}_{{{{\rm{LPN}}}}_{{{\rm{left-over}}}}}(f)}\approx 8\pi \delta f\sqrt{{S}_{{{\rm{LPN}}}}(f)}\,,$$

where *δ* is the error in how well the time of flight for light between the two satellites is known. With GPS satellite positioning on the order of 5 mm^34,35^, *δ* = 5 mm/*c* ≈ 10^−10^ s. Hence, at frequencies close to 10^−2^ Hz, the laser phase noise can be cancelled by approximately 10 orders of magnitude. This is more than sufficient to ensure the laser phase noise is no longer a dominant noise source. However, the signal-to-noise ratio (SNR) of the remaining noise sources is influenced by TDI. As a result of applying TDI the signal is affected such that

12 $$\frac{\left|{a}_{{{\mbox{R,TDI}}}}(f)\right|}{\left|{a}_{{{\mbox{R}}}}(f)\right|}=4\left|\,{{\mbox{sin}}}\,\left(\frac{2\pi f}{{f}_{L}}\right)\right|\left|\,{{\mbox{sin}}}\,\left(\frac{\pi f}{{f}_{c}}\right)\right|\,.$$

Similarly, the remaining noise sources are affected in the following manner

13 $$\frac{\sqrt{{S}_{{{\mbox{AN,TDI}}}}(f)}}{\sqrt{{S}_{{{\mbox{AN}}}}(f)}}=2\sqrt{2}\left|\,{{\mbox{sin}}}\,\left(\frac{\pi f}{{f}_{c}}\right)\right|\,,$$

and

14 $$\frac{\sqrt{{S}_{{{\mbox{QN,TDI}}}}(f)}}{\sqrt{{S}_{{{\mbox{QN}}}}(f)}}=2\sqrt{2}\left|\,{{\mbox{sin}}}\,\left(\frac{\pi f}{{f}_{c}}\right)\right|\,,$$

as discussed in Supplementary Note 8. Thus, the ratio of the SNR with TDI to the SNR without TDI for both the quantum noise and the accelerometer noise is $\sqrt{2}\left|\,{{\mbox{sin}}}\,\left(2\pi f/{f}_{L}\right)\right|$. The change in SNR as a function of frequency for both quantum noise and accelerometer noise is shown in Fig. 3b. Importantly, the SNR for the remaining noise sources is enhanced by $\sqrt{2}$ when *f* = *v*_0_/2*L* ≈ 1 × 10^−2^ Hz, which is very close to the frequency of interest where the point mass gravitational signal is maximal. The reason for the SNR enhancement is that we are now measuring the phase shift between two pairs of satellites, instead of one pair as in the original mission. However, the SNR is degraded at certain frequencies, near the nodes in Fig. 3b, and one important implication of this is that TDI is most beneficial when the laser phase noise is the dominant noise source. A comparison of the signal and total noise spectra, both with and without TDI, is shown in Fig. 6. This shows the SNR enhancement that TDI can offer over a current GRACE style mission. Note that for real satellite geodesy missions, the frequencies of interest cover a broad range, at some of which, TDI will degrade the SNR with respect to the remaining noises. A more complete analysis will be required to determine the utility of TDI for real world satellite geodesy.

### Minimum detectable mass

It is to be expected that TDI can aid satellite geodesy as one of the major noise sources is removed without the signal being totally compressed. This can be made rigorous by considering the minimum detectable mass defined as^31^

15 $${M}_{{{\rm{min}}}}=\frac{3}{\sqrt{4\int\nolimits_{0}^{\infty }\frac{{\left|{a}_{{{\rm{R}}}}(f)/M\right|}^{2}}{{S}_{{{\rm{T}}}}(f)}df}}\,,$$

where *S*_T_ is the total noise spectrum given by

16 $${S}_{{{\mbox{T}}}}={S}_{{{\mbox{AN}}}}+{S}_{{{\mbox{LPN}}}}+{S}_{{{\mbox{QN}}}}\,.$$

This is the minimum mass which corresponds to a SNR of at least 3, which intuitively represents the smallest possible mass our system can detect. We now define the following quantity as the TDI gain

17 $${{{{\mathcal{G}}}}}_{TDI}=\frac{{M}_{{{\mbox{original}}}}}{{M}_{{{\mbox{TDI}}}}}\,,$$

where *M*_original_ is the minimum detectable mass in the original scheme without TDI, formation ${\alpha }_{2}^{O}$, and *M*_TDI_ is the minimum detectable mass with TDI, formation ${\alpha }_{3}^{T}$. Intuitively the TDI gain tells us how many times smaller a mass can be detected with TDI than without.

**Fig. 4:**
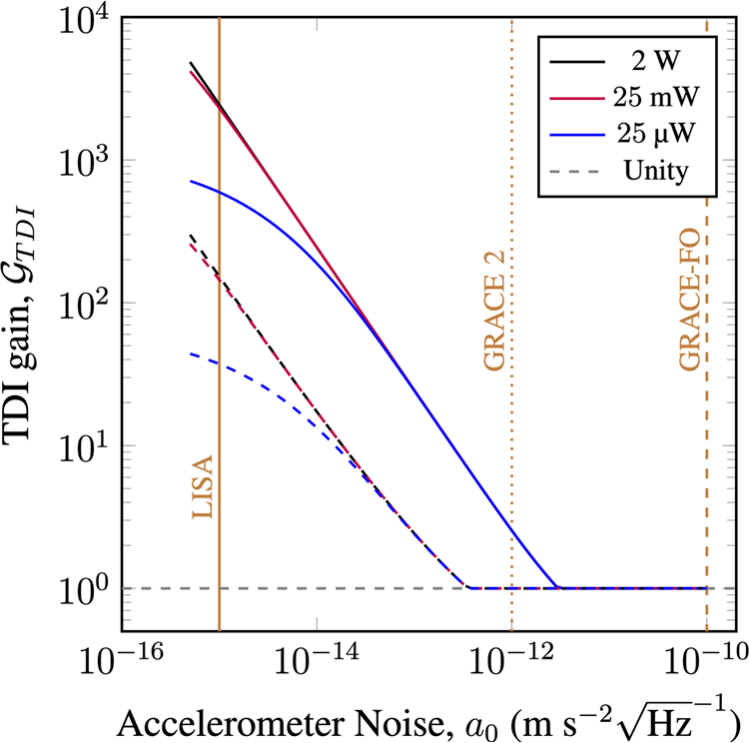
Point mass sensitivity enhancement due to TDI. Plotted is the TDI gain as a function of accelerometer instrument noise for the two different laser phase noise performances, the GRACE-FO laser phase noise requirement, Eq. (4) (solid lines, ${x}_{c}=8\,{{\mbox{nm}}}{\sqrt{{{\mbox{Hz}}}}}^{-1}$) and the actual laser phase noise performance, Eq. (5) (dashed lines, *x*_*T*_ = 1 × 10^−15^). The TDI gain increases with decreasing accelerometer instrument noise as at lower accelerometer instrument noise levels the cancellation of the laser phase noise is more impactful. However, this does not increase indefinitely as eventually the quantum noise limit is reached. Three different quantum noise levels are considered, corresponding to transmitted powers of 2 W, 25 mW and 25 *μ*W, at a satellite separation of *L*_12_ = *L*_23_ = 200 km with a receiving aperture radius of 5 cm. The satellite orbital height is *h* = 500 km. The vertical dashed, dotted and solid brown lines show the projected accelerometer instrument noise for the GRACE-FO mission, the next GRACE mission (GRACE 2) and the LISA mission respectively. At large accelerometer noises the TDI gain is never less than 1, because TDI is a non-destructive measurement.

With realistic future accelerometer instrument noise levels, TDI has the potential to significantly reduce the minimum detectable mass, as shown in Fig. 4. At high accelerometer instrument noises the laser phase noise is not important and so TDI does not offer any improvement. However, as TDI is a non-destructive measurement we can simply choose not to use TDI in postprocessing. With reducing accelerometer instrument noise the TDI gain increases, until when the accelerometer instrument noise is sufficiently low, quantum noise becomes the major noise source and so the advantage flattens off. At very low values of *a*_0_, when quantum noise starts to dominate there is an advantage to increasing the laser power. Equivalently, this advantage can be obtained from increasing the receiving aperture size, or any technique to reduce quantum noise, such as optical squeezing^36^. For sufficiently small accelerometer and quantum noise levels, the left-over laser phase noise after TDI may become the limiting factor again. The sensitivity gain offered by TDI is very close to being achievable with today’s technology, with the accelerometers of past and planned missions having *a*_0_ values in the range ${a}_{0}\approx 1\times 1{0}^{-12}\,{{\mbox{m}}}{{{\mbox{s}}}}^{-2}{\sqrt{{{\mbox{Hz}}}}}^{-1}\to {a}_{0}\approx 1\times 1{0}^{-15}\,{{\mbox{m}}}{{{\mbox{s}}}}^{-2}{\sqrt{{{\mbox{Hz}}}}}^{-1}$^37–39^.

**Fig. 5:**
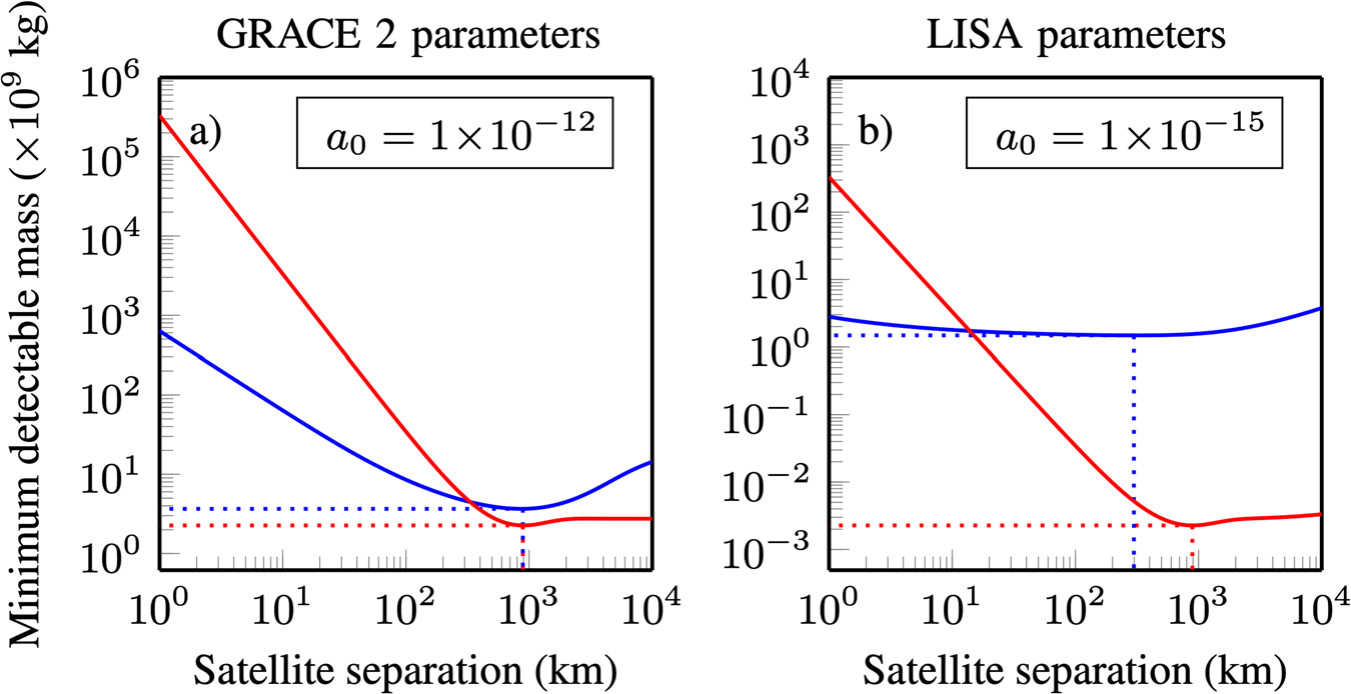
Optimal satellite separation for point mass sensitivity. Shown is the point mass sensitivity both with (red lines) and without (blue lines) TDI for accelerometer noises of ${a}_{0}=1\times 1{0}^{-12}\,{{\mbox{m}}}{{{\mbox{s}}}}^{-2}{\sqrt{{{\mbox{Hz}}}}}^{-1}$ (**a**) and ${a}_{0}=1\times 1{0}^{-15}\,{{\mbox{m}}}{{{\mbox{s}}}}^{-2}{\sqrt{{{\mbox{Hz}}}}}^{-1}$ (**b**) as a function of the satellite separation. Laser thermal noise is *x*_*T*_ = 1 × 10^−15^ and satellite orbital height is *h* = 500 km. The optimal satellite separation is that which minimises the minimum detectable mass, and is different for different mission configurations.

In Fig. 5 the minimum detectable mass as a function of satellite separation is shown for geodesy both with and without TDI. The optimal satellite separation (that which minimises the minimum detectable mass) differs depending on the strategy employed. Without TDI, improvements in the accelerometer instrument noise produce only marginal improvements in sensitivity. However, the same improvement combined with TDI can vastly improve sensitivity. Without TDI, the upgrade of the GRACE-FO accelerometer from ${a}_{0}=1\times 1{0}^{-12}\,{{\mbox{m}}}{{{\mbox{s}}}}^{-2}{\sqrt{{{\mbox{Hz}}}}}^{-1}$ to ${a}_{0}=1\times 1{0}^{-15}\,{{\mbox{m}}}{{{\mbox{s}}}}^{-2}{\sqrt{{{\mbox{Hz}}}}}^{-1}$ induces a very minor improvement. In contrast, the use of TDI in the same conditions leads to an improvement of nearly three orders of magnitude, with the minimum detectable mass being almost 1 × 10^6^ kg. For perspective, this mass is equivalent to a change in water or ice levels almost as small as 1 mm over a 1 km^2^ area. However, we note again that these calculations are based on the 1D point mass model and so are not directly related to actual satellite geodesy missions. Additionally, if the laser phase noise and accelerometer noise are sufficiently reduced, other noise sources may start to dominate^18,19^. In Supplementary Note 9 similar calculations are presented for a range of satellite orbital heights.

The current GRACE-FO mission uses an optical cavity to achieve an improved frequency stability. We now compare the point mass sensitivity, both with and without TDI, in terms of requisite laser stability. The leftover laser phase noise is calculated assuming the satellite positions are known to within 5 mm. For ${a}_{0}=2\times 1{0}^{-13}\,{{\mbox{m}}}{{{\mbox{s}}}}^{-2}{\sqrt{{{\mbox{Hz}}}}}^{-1}$, to achieve the same point mass sensitivity as is provided by using TDI and a laser with *x*_*T*_ ≈ 1 × 10^−12^, without using TDI requires a laser with three orders of magnitude more stability, *x*_*T*_ = 1 × 10^−15^. However, as above, for real-world geodesy this relaxation in laser stability may not be true owing to the more complex frequency dependence of the gravitational signal. Specifically, for recovering signals at low frequencies where TDI degrades the SNR, this relaxation in laser stability would not be possible.

### Accelerometer noise

The purpose of the accelerometer is to measure the non-gravitational acceleration as accurately as possible so that it can be removed from the measurement while adding the minimum amount of noise. Ultimately however, the accelerometer will always add some noise. We now show that through precise satellite engineering and formation flying, the line-of-sight non-gravitational acceleration can be removed from the measurement without using an accelerometer. This eliminates a major noise source, accelerometer instrument noise. The principle behind this is that the non-gravitational forces acting on the satellites consist of a stationary and a non-stationary component. These non-gravitational forces then give rise to non-gravitational accelerations, which have a stationary, ${a}_{\,{{\mbox{s}}}}^{{{\mbox{ng}}}\,}$ and a non-stationary, ${a}_{\,{{\mbox{ns}}}}^{{{\mbox{ng}}}\,}$, component. Stationarity here refers to temporal stationarity. If the leading satellite is at position *x* at time *t* and the trailing satellite reaches *x* at a time *t* + Δ*t*, then the stationary non-gravitational accelerations will be common to both satellites, ${a}_{\,{{\mbox{s}}}}^{{{\mbox{ng}}}}(x,t)={a}_{{{\mbox{s}}}}^{{{\mbox{ng}}}\,}(x,t+{{\Delta }}t)$ and the non-stationary non-gravitational forces will differ ${a}_{\,{{\mbox{ns}}}}^{{{\mbox{ng}}}}(x,t)\,\ne\, {a}_{{{\mbox{ns}}}}^{{{\mbox{ng}}}\,}(x,t+{{\Delta }}t)$.

The stationary non-gravitational accelerations experienced by all satellites will be the same provided they have the same mass and identical aerodynamicity. The similarity of the non-stationary non-gravitational accelerations experienced by each satellite, i.e., how much ${a}_{\,{{\mbox{ns}}}}^{{{\mbox{ng}}}\,}(x,t)$ and ${a}_{\,{{\mbox{ns}}}}^{{{\mbox{ng}}}\,}(x,t+{{\Delta }}t)$ differ, is correlated to the satellite separation. The further the satellites are apart the more the non-stationary component will have changed by the time it takes the trailing satellite to reach the position of the leading satellite.

### Six satellite differential mass formation flying with TDI

We now turn to the satellite combinations presented in Fig. 2 c and d, formations ${\alpha }_{3,DM}^{T}$ and ${\alpha }_{6,DM}^{T}$ respectively. Neither of these combinations require an accelerometer and so do not introduce any accelerometer instrument noise. Instead these formations rely on satellites of precisely known, but different masses which will experience different non-gravitational accelerations. Assuming identical aerodynamicity, the same non-gravitational force acting on two satellites, one with twice the mass of the other, will result in twice the non-gravitational acceleration for the lighter satellite. This principle allows common mode non-gravitational accelerations to be removed from the measurement, and is the reason an accelerometer is no longer required. Formation ${\alpha }_{3,DM}^{T}$ relies on only three satellites separated by distances on the order of hundreds of kilometers. As the satellites are so distant from one another, only the stationary component of the non-gravitational accelerations will be common to all three satellites, allowing this to be removed from the measurement. This formation does not allow the non-stationary component of the non-gravitational acceleration to be removed. As such, formation ${\alpha }_{3,DM}^{T}$ performs worse than the current GRACE-FO mission with realistic parameters and so we defer further discussion of this to Supplementary Note 10.

Formation ${\alpha }_{6,DM}^{T}$ is more promising as it is, in theory, able to *completely remove laser phase noise and accelerometer noise*. This scheme works by making two independent sets of measurements with effectively the same laser. The scheme is broken into 3 pairs of different mass satellites, where, as before the different pairs will be separated by hundreds of kilometers. However, the satellites within each pair are required to stay as close as possible to each other. The two satellites in each pair, which are of mass M_*S*_ and 2M_*S*_, are called A and B satellites respectively. Owing to the different masses, the B satellites will experience half the non-gravitational accelerations the A satellites experience. Importantly, as each A-B pair is close to each other, they will experience almost the same stationary and non-stationary non-gravitational forces. This allows for the near-perfect removal of non-gravitational accelerations. Owing to the different non-gravitational accelerations experienced, thruster movements will be required to keep each pair close to each other. It is only by having two satellites with different masses close to each other that the non-stationary component of the non-gravitational accelerations can be removed.

Satellites in this scheme are denoted *S*_*i*,*j*_, with *i* ∈ {*A*, *B*} denoting whether the satellite is the heavier (B) or lighter (A) of this particular pair and *j* ∈ {1, 2, 3} denoting which pair of satellites we refer to (1 being the leading satellite and 3 the trailing satellite). The laser on satellite *S*_*B*2_ is sent to satellite *S*_*A*2_, and through a short delay fibre on satellite *S*_*B*2_, it can be arranged that both satellites are using effectively the same laser. This light is sent to the outer satellites and reflected back to the middle satellites where two measurements are made by each of the middle A-B pair. Satellite *S*_*A*2_ measures

18 $$\begin{array}{l}2{\hat{\phi }}_{A21(3)}(t)={{\mbox{}}}2{\phi }_{21(3)}^{{{\mbox{g}}}\,}(t)+C(t-2{\tau }_{21(3)})-C(t)\\\qquad\qquad\qquad\,\,+\,Q{N}_{1(3)A}(t)-2{\phi }_{1(2)}^{\,{{\mbox{ng}}}}(t)+2{\phi }_{2(3)}^{{{\mbox{ng}}}\,}(t),\end{array}$$

and *S*_2*B*_ measures

19 $$\begin{array}{l}2{\hat{\phi }}_{B21(3)}(t)=\,{{\mbox{}}}2{\phi }_{21(3)}^{{{\mbox{g}}}\,}(t)+C(t-2{\tau }_{21(3)})-C(t)\\ \qquad\qquad\qquad\,\,+\,Q{N}_{1(3)B}(t)-{\phi }_{1(2)}^{\,{{\mbox{ng}}}}(t)+{\phi }_{2(3)}^{{{\mbox{ng}}}\,}(t)\,,\end{array}$$

where *QN*_*ij*_(*t*) denotes the quantum noise at time *t* on satellite *S*_*j*2_ for light received from satellite *S*_*ji*_. These measurements can be combined to give two total measurement terms with no accelerometer noise, $2{\phi }_{T21}(t)=4{\hat{\phi }}_{B21}(t)-2{\hat{\phi }}_{A21}(t)$ and $2{\phi }_{T23}(t)=4{\hat{\phi }}_{B23}(t)-2{\hat{\phi }}_{A23}(t)$. The laser phase noise can then be removed from these two measurements using the same TDI combination discussed earlier. We re-emphasise that in principle this combination requires *no on-board accelerometer* and *no optical cavity* provided the satellites can be flown with sufficient accuracy.

With an orbital height of *h* = 500 km and satellite separation of *L* = 200 km this scheme can achieve a minimum detectable mass of 9 × 10^4^ kg, assuming perfect accelerometer noise cancellation, laser phase noise cancellation to within 5 mm and a transmitted laser power of 2 W. This is approximately 6 orders of magnitude better than the current GRACE mission (3 × 10^11^ kg, assuming *x*_*T*_ = 1 × 10^−15^), more than 4 orders of magnitude better than the current mission with an improved accelerometer, ${a}_{0}=1\times 1{0}^{-12}\,{{\mbox{m}}}\,{{{\mbox{s}}}}^{-2}{\sqrt{{{\mbox{Hz}}}}}^{-1}$, (5 × 10^9^ kg) and approximately 3 orders of magnitude better than the TDI combination with an ambitious level of accelerometer instrument noise, ${a}_{0}=1\times 1{0}^{-14}\,{{\mbox{m}}}\,{{{\mbox{s}}}}^{-2}{\sqrt{{{\mbox{Hz}}}}}^{-1}$, (9 × 10^7^ kg). Note that the minimum detectable mass presented here for the current GRACE mission (3 × 10^11^ kg) is different to the value quoted by Spero^31^, as we use a slightly different definition of the signal strength and a different satellite separation. The potentially huge improvement in sensitivity makes the significant technological challenge of implementing this scheme one worth considering.

In reality, the A-B satellites in each pair will not be in exactly the same position, and the separation of each pair will drift over time. The further apart the satellites in each A-B pair are, the larger the difference in the non-stationary component of the non-gravitational force experienced will be. The effect of this is that the non-gravitational acceleration cancellation will not be perfect. However, even if each pair of satellites cannot be made to fly exactly alongside one another, some cancellation can still be achieved. An approximate model for the difference in non-gravitational acceleration in the along-track direction experienced by a pair of satellites separated by 200 km, after data transplanting (a technique used to estimate the non-gravitational accelerations of one satellite using accelerometer data from the other satellite) is

20 $${a}^{{{\mbox{ng}}}}(f,200\,{{\mbox{km}}}\,)\approx \frac{{a}_{n}}{{\left[1+{\left(\frac{f}{{f}_{n}}\right)}^{2}\right]}^{3}}\,,$$

where ${a}_{n}=2\times 1{0}^{-8}{{{\mbox{m s}}}}^{-2}{\sqrt{{{\mbox{Hz}}}}}^{-1}$ and *f*_*n*_ = 3 × 10^−2^ Hz^40^. We make the assumption that the difference in non-gravitational accelerations will scale linearly with distance, such that ${a}^{{{\mbox{ng}}}}(f,x)={a}^{{{\mbox{ng}}}}(f,200{{\mbox{km}}}\,)\cdot \left(x/200\,{{\mbox{km}}}\,\right)$. As the spectrum in Eq. (20) is obtained when the non-gravitational data from one satellite has been transplanted, which is not the case with our scheme, the true differential non-gravitational accelerations will be larger than those predicted by this model. However, as the satellites become closer the difference between transplanting and not transplanting becomes smaller. In an ideal implementation of our scheme each satellite-pair will be separated by no more than a few metres, hence this difference would be small. After TDI the leftover non-gravitational accelerations are scaled by a factor of $2\sqrt{2}\left|\,{{\mbox{sin}}}\,\left(\pi f/{f}_{c}\right)\right|$. This approximate model can be used to place bounds on the performance of this scheme.

**Fig. 6:**
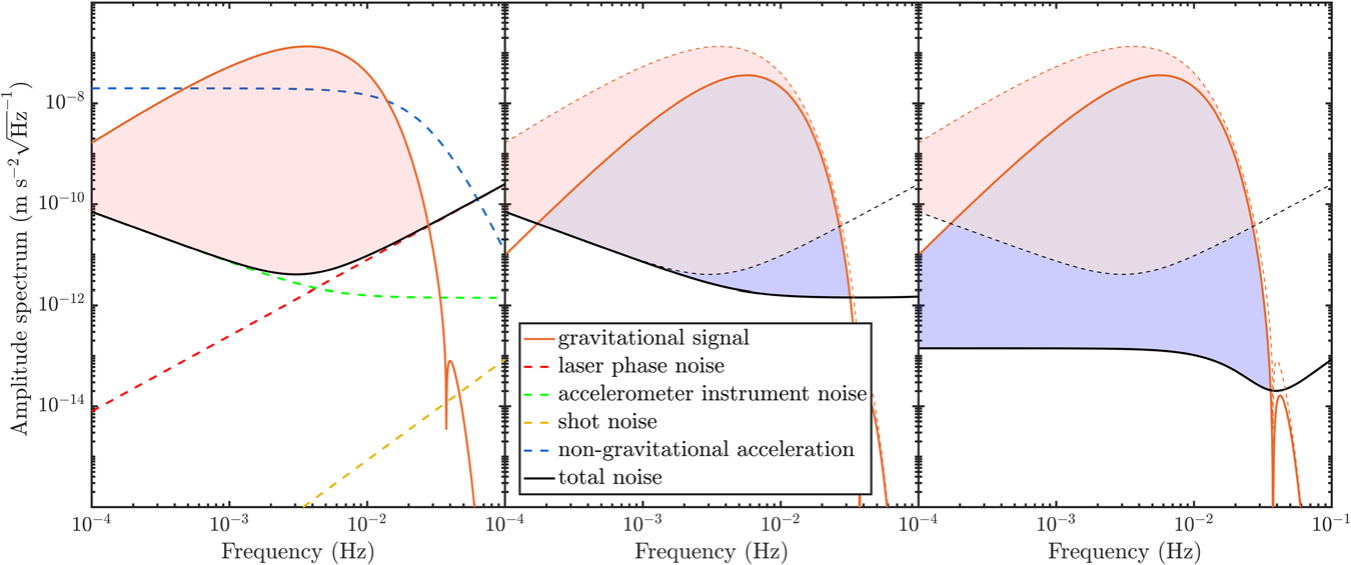
Noise spectrum analysis of proposed satellite geodesy missions. **a** Signal and noise spectra for a GRACE-FO-like, two-satellite mission, formation ${\alpha }_{2}^{O}$. The gravitational signal corresponds to a 1 × 10^13^ kg point mass, satellite separation of *L* = 200 km and satellite orbital height of *h* = 500 km (Eq. (1)), accelerometer instrument noise corresponds to ${a}_{0}=1\times 1{0}^{-12}\,{{\mbox{m}}}{{{\mbox{s}}}}^{-2}{\sqrt{{{\mbox{Hz}}}}}^{-1}$ (Eq. (2)), laser phase noise corresponds to *x*_*T*_ = 1 × 10^−15^ (Eq. (5)), the quantum noise level is calculated by considering the diffraction limits set by a 25 cm receiving aperture radius and 25 mW of initial optical power (Eq. (6)) and the non-gravitational accelerations are those from Eq. (20). The region highlighted in red corresponds to frequencies where the signal is above the noise floor, i.e., the region which contributes most to enhancing the signal-to-noise ratio. **b** Signal and total noise spectrum for a formation ${\alpha }_{3}^{T}$ mission, with three satellites and TDI being employed. The gravitational signal after TDI is given by Eq. (10). The dashed orange and black lines correspond to the gravitational signal and total noise from the ${\alpha }_{2}^{O}$ mission. The region highlighted in red corresponds to regions which can only be accessed by the ${\alpha }_{2}^{O}$ mission, the region highlighted in blue shows the new region which can be accessed by the ${\alpha }_{3}^{T}$ mission and the mauve region in between is accessible for both missions. As TDI is a non-destructive measurement the region where the SNR> 1 for the ${\alpha }_{2}^{O}$ mission is still accessible. The increase in the size of the shaded region highlights the benefit of TDI in this instance. Satellite positions are assumed to be known to within 5 mm. The spectra corresponding to scheme ${\alpha }_{3}^{T}$ with TDI are rescaled by $1/(2\sqrt{2}\left|\,{{\mbox{sin}}}\,(\pi f/{f}_{c})\right|)$ so that quantum noise and accelerometer noise are unaffected by TDI. **c** Signal and total noise spectrum for a formation ${\alpha }_{6,DM}^{T}$ style mission, with six different mass satellites and TDI being employed. Again the blue region corresponds to the benefit of this scheme, the region which cannot be accessed by the ${\alpha }_{2}^{O}$ mission. Each A-B satellite pair is assumed to fly within 1 m of each other. The spectra corresponding to scheme ${\alpha }_{6,DM}^{T}$ with TDI are rescaled by $1/(2\sqrt{2}\left|\,{{\mbox{sin}}}\,(\pi f/{f}_{c})\right|)$. For all of the plots the gravitational signals have units ms^−2^ Hz^−1^.

In order for this technique to outperform TDI alone this cancellation must be below the projected accelerometer noise. The non-gravitational acceleration can be further reduced if the overall drag of the satellites is reduced, as would be the case by transitioning to CubeSats, or changing orbital height. Imperfect satellite flying in this scheme means that the required TDI combination becomes slightly more complex, with the necessary combination shown in Supplementary Note 11. If each A-B pair of satellites can be flown within 1 m of each other, while reducing the total drag of each satellite by a factor of ten compared to the current GRACE mission, then this technique for accelerometer noise cancellation is equivalent to having an accelerometer with ${a}_{0}\approx 5\times 1{0}^{-15}\,{{\mbox{m}}}\,{{{\mbox{s}}}}^{-2}{\sqrt{{{\mbox{Hz}}}}}^{-1}$ in terms of minimum detectable mass. Thus, although we are using a simplified model of the non-gravitational accelerations, it is possible that this scheme can yield significant improvements with future technologies. Figure 6c compares the signal and total noise spectra of a GRACE-FO-like mission in its present state with all noise sources to our proposed implementation of this six-satellite scheme, formation ${\alpha }_{6,DM}^{T}$, at an orbital height of 500 km, satellite separation of 200 km between trailing satellites and 1 m between each A-B pair of satellites.

The enhancement discussed in this section relies on a number of simplifying assumptions which will not be true in practice, regarding the differential non-gravitational accelerations. For example, the satellite masses will change over the course of the mission, thruster firings will affect the satellite accelerations and the satellites would not have identical aerodynamicity. Our scheme is only capable of removing line-of-sight non-gravitational accelerations, hence for real world geodesy, accelerometers may still be necessary to remove 3D non-gravitational accelerations. Additionally, there are significant technological hurdles to overcome before a mission configuration as complex as this can be used in reality. Finally, there are many practical issues with flying each A-B satellite pair close to each other, such as the risk of collision and the fact that the satellites will drift apart and follow slightly different orbits. These imperfections will manifest as ranging errors. Nevertheless, with further development, the principle behind this configuration may one day be of great use to geodesy missions. For instance, it may be possible to avoid some of these difficulties by replacing each satellite pair with a single satellite containing two different mass test masses in freefall.

### Quantum limited satellite geodesy

Having suggested schemes for reducing the laser phase noise and accelerometer noise, we now turn our attention to reducing the quantum noise limit of satellite-based geodesy. The current GRACE-FO mission is not quantum noise limited and so does not reach the fundamental quantum interferometry bound^41^. However, future satellite missions may one day approach the quantum limit. For example, the quantum noise limit can be reached either with instrument enhancement, i.e., improvements in optical cavity stability and accelerometer instrument noise, or through the multi-satellite formations presented above. One way to reduce quantum noise is using squeezed light^42–44^, which would reduce the quantum noise term *δ*_*QN*_ in Eq. (6), by a factor *e*^*r*^, where *r* is the squeezing level. Thus, from Eq. (15) we can expect that squeezing can provide an enhancement of up to *e*^*r*^ in terms of minimum detectable mass.

**Fig. 7:**
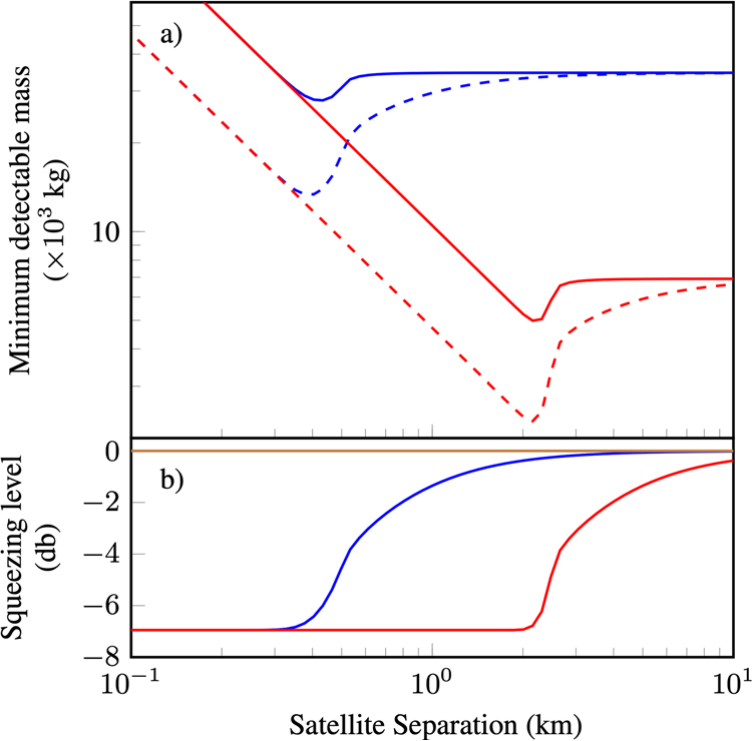
Minimum detectable mass for quantum noise limited geodesy. **a** The benefit from using squeezed light is obtained within the satellite separation where diffraction losses are not significant. This distance can be extended by increasing the aperture of the receiving optics. The blue and red lines correspond to receiving optics with aperture radius of 5 and 25 cm, respectively. Dashed lines indicate the point mass sensitivity using squeezed light. Parameters used are input power *P*_0_ = 25 mW and initially 7 dB of pure squeezing. **b** Effective squeezing level as a function of satellite separation. Brown line corresponds to the quantum noise level.

Interestingly, depending on how the quantum noise limited regime is reached, the optimal satellite separation is different. If the quantum noise limited regime is reached through enhancements in instrument noise there is a new regime which is optimal for satellite geodesy. When quantum noise limited, a larger satellite separation increases the signal strength but also increases the noise floor as the received optical power and squeezing level are reduced. The optimal satellite separation is that which minimises this trade-off, as shown in Fig. 7. The smallest minimum detectable mass now occurs at the point where diffraction loss first becomes noticeable, which for 25 cm receiving apertures is at approximately 2 km. This is not at all obvious as at greater satellite separations the signal strength is much larger. However, by transitioning to a mission with reduced satellite separation, the benefits of squeezing and a greater received optical power compensate for the reduced signal strength. This was verified with a full 3D numerical simulation of satellites flying in the Earth’s gravitational field when quantum noise limited, shown in Supplementary Note 12. It should be noted that 25 cm radius receiving aperture optics would be considerably more expensive than what is presently used (for reference the LISA mission plans to use a 15 cm radius telescope^8^).

Alternatively, if the quantum noise limit is reached by multi-satellite formation flying combined with TDI, the optimal satellite separation can be much greater, extending far beyond the separation where squeezing stops being useful due to excessive propagation loss. This is because TDI will reduce the signal strength more as the two measurements become more correlated, which happens when the satellites are closer as the gravitational field experienced is more similar. This was also verified with our 3D model, discussed further in Supplementary Note 12. In addition to the use of squeezing, several other quantum techniques, including optical delay lines and distributing multi-mode entangled states between satellites were investigated and found to have varying degrees of utility in the quantum noise limited regime. This will be the subject of future research.

For 200 km satellite separation, in order to be in the quantum noise limited region, significant technological progress is necessary, requiring a laser thermal noise of *x*_*T*_ ≈ 5 × 10^−20^ and an acceleration white noise of ${a}_{0}\approx 2\times 1{0}^{-16}{{{\mbox{m s}}}}^{-2}{\sqrt{{{\mbox{Hz}}}}}^{-1}$ as shown in Supplementary Note 13. Furthermore, sub-hertz squeezing would be necessary if squeezed light is to be useful for geodesy. One might imagine that by transitioning to an alternate mission where the satellites are much closer (on the order of metres) we may enter a regime where quantum noise is the limiting factor as the laser phase noise will be greatly reduced. However, this is not the case as at smaller satellite separations the quantum noise is also greatly reduced due to the detection of more optical power. The high frequency roll-off in the gravitational signal makes the quantum noise limited regime difficult to reach for typical satellite parameters owing to the different frequency dependence of laser phase noise and quantum noise. The only way to get around the high frequency roll-off is by transitioning to lower orbital heights. In Supplementary Note 13 we propose a new type of mission which operates in this regime. Such a mission is impossible for mapping the Earth’s gravitational field, but may find use for mapping the gravitational field of other astronomical bodies^45^. This type of mission appears to be in the quantum noise limited regime, allowing for squeezed light enhanced geodesy. However, quantum noise can also be further reduced by increasing the optical power. This pushes the need for squeezed light even further away. Increasing the optical power is currently a less technically challenging method of reducing the quantum noise than generating squeezed light in space. Nevertheless, the techniques presented in this section may someday be useful for satellite geodesy. We liken this to Carlton Caves’ original proposal to use squeezed light in the search for gravitational waves^16^, which after decades of technological progress will reveal the quantum noise limit of an instrument, as in LIGO^46^.

## Discussion

In this paper several techniques have been presented which can be applied to satellite geodesy to enhance the point mass sensitivity. The potential improvements we have proposed rely on some simplifying assumptions, namely circumscribing the analysis to point mass sensitivity and measurement noise. However, the proposed techniques show great promise which may motivate further studies with fewer assumptions. We have shown that time delay interferometry can offer significant benefits, in terms of the minimum detectable point mass. Time delay interferometry can be implemented with current technology and would be a useful technology demonstration for LISA. With a LISA-grade accelerometer, time delay interferometry can offer almost 3 orders of magnitude improvement in point mass sensitivity. Precisely controlled multi-satellite formations were presented which can remove accelerometer noise and laser phase noise simultaneously. Importantly, these formations do not require on-board accelerometers nor optical cavities. Finally, the possibility of reducing quantum noise through the injection of squeezing has been studied. Although squeezed light has the potential to improve satellite geodesy, significant technological enhancements are required before this becomes relevant. Nevertheless, we anticipate that the techniques presented here will have a crucial role to play in the enhancement of satellite geodesy in the future.

### Reporting summary

Further information on research design is available in the Nature Research Reporting Summary linked to this article.

## Supplementary information

Supplementary Information

Reporting Summary

## Data Availability

The data that supports the findings of this study are available from the corresponding author upon reasonable request.

## Code availability

The code that supports the findings of this study are available from the corresponding author upon reasonable request.
