# Supplementary material for: Enhancing the precision limits of interferometric satellite geodesy missions
Source: NPJ Microgravity. 2022 Jun 8;8:21. doi: 10.1038/s41526-022-00204-9 (PMC9177761; doi:10.1038/s41526-022-00204-9)
Supplement: Supplementary file 1 — Supplementary Information [file 41526_2022_204_MOESM1_ESM.pdf]

Supplementary information : Enhancing the precision limits of interferometric satellite geodesy missions

Lorcán O. Conlon,[1,*] Thibault Michel,[1] Giovanni Guccione,[1]
Kirk McKenzie,[2,3] Syed M. Assad,[1,4] and Ping Koy Lam[1,4]

[1] *Centre for Quantum Computation and Communication Technology, Department of Quantum Science, Australian National University, Canberra, ACT 2601, Australia.*
[2] *Centre for Gravitational Astrophysics (CGA), Research School of Physics, The Australian National University, Canberra ACT 2601, Australia*
[3] *ARC Centre of Excellence for Gravitational Wave Discovery (OzGrav), Research School of Physics, The Australian National University, Canberra ACT 2601, Australia*
[4] *School of Physical and Mathematical Sciences, Nanyang Technological University, Singapore 639673, Republic of Singapore*

(Dated: April 11, 2022)

Supplementary Note 1 . FOURIER DOMAIN TWO SATELLITE ACCELERATION RANGE SIGNAL

We consider a satellite travelling with an initial velocity v_0 at a height h above a plane. There is a point mass M on the plane, which at time, $t = 0$, is a horizontal distance x from the satellite, so that the total distance from the satellite to the mass is $\sqrt{h^2 + x^2}$. Assuming that the along track position is not affected significantly by the gravitational attraction, $x = v_0 t$, the acceleration in the along track direction for a single satellite in the time domain is given by

$$a(t) = -\frac{GMtv_0}{(h^2 + t^2 v_0^2)^{3/2}} \;, \tag{1}$$

where $G = 6.67 \times 10^{-11}$ m^{3}kg^{-1}s^{-2} is the gravitational constant. Converting to the frequency domain gives

$$a(f) = -\frac{GMv_0}{h^3} \int_{-\infty}^{\infty} \frac{e^{-2\pi \mathrm{i} f t} t}{(1 + \frac{t^2 v_0^2}{h^2})^{3/2}} dt \;. \tag{2}$$

Using the substitution $u = tv_0/h$, this can be written

$$a(f) = \frac{-GM}{hv_0} \int_{-\infty}^{\infty} \frac{e^{-2\pi \mathrm{i} f \frac{h}{v_0} u} u}{(1 + u^2)^{3/2}} du \;. \tag{3}$$

Through the product rule (using $b = e^{-2\pi \mathrm{i} f u h / v_0}, dc = u/(1 + u^2)^{3/2}$) this becomes

$$a(f) = \frac{-GM}{hv_0} \left[\frac{-e^{-2\pi \mathrm{i} f \frac{h}{v_0} u}}{(1 + u^2)^{1/2}} \Bigg|_{u=-\infty}^{u=\infty} - \int_{-\infty}^{\infty} \frac{2\pi \mathrm{i} f \frac{h}{v_0} e^{-2\pi \mathrm{i} f \frac{h}{v_0} u}}{\sqrt{1 + u^2}} du \right] . \tag{4}$$

The first term is zero and the second term can be identified as a multiple of the modified Bessel function of the second kind, order 0, K_0, giving

$$a(f) = \frac{4\pi \mathrm{i} f GM}{v_0^2} K_0 \left(\frac{2\pi f}{f_h} \right) \;, \tag{5}$$

* lorcan.conlon@anu.edu.au

where $f_h = v_0/h$. This is the acceleration of a single satellite in the frequency domain. For satellite geodesy missions we are interested in how the range between a pair of satellites changes in time and so we consider the range acceleration.

The range acceleration is the differential acceleration of the two satellites, obtained by subtracting one signal from the other. The effect of subtracting one signal from another is to multiply the signal by a sin term.

$$\begin{aligned} S(t) &= a(t) - a(t-\tau) \\ \mathcal{S}(f) &= \mathcal{F}(S(t)) = a(f)(1 - e^{-2\pi i f \tau}) \\ |\mathcal{S}(f)| &= |a(f)||e^{-\pi i f \tau}||(e^{\pi i f \tau} - e^{-\pi i f \tau})| \\ |\mathcal{S}(f)| &= |a(f)||2\,\sin(\pi f \tau)|\ . \end{aligned} \tag{6}$$

For a GRACE-like mission one satellite follows another along a very similar trajectory, corresponding to a delay of $\tau_S = L/v_0$, where L is the satellite separation and the subscript S denotes that this time of flight corresponds to the satellite velocity. Thus, for a single satellite pair the range acceleration in the frequency domain is

$$|a_R(f)| = \frac{8\pi f GM}{v_0^2} K_0\left(\frac{2\pi f}{f_h}\right)\left|\sin\left(\frac{2\pi f}{f_L}\right)\right|\ , \tag{7}$$

where $f_L = 2v_0/L$. As the current GRACE-FO mission measures twice the phase shift between the two satellites, we scale this expression by a factor of 2 in Eq. (1) in the main text. There are nulls in the signal at certain frequencies, corresponding to $f = \mathrm{n}v_0/L$, where n is any integer. These nulls can be seen in Fig. 6 of the main text. Intuitively, any signal with a period equal to the satellite separation time, τ_S, will not be observed in the ranging signal as it will affect both satellites in the same manner. The same is true for any period which is an integer fraction of τ_S. This model was presented in its entirety in Ref. [1].

Supplementary Note 2 . LASER PHASE NOISE SPECTRUM

We note here why the laser phase noise scales linearly with distance. From Eq. (3) in the main text, the laser phase noise in the current GRACE-FO measurement is

$$C_{\mathrm{tot}}(t) = C_1(t) - C_1(t - 2\tau_{12})\ , \tag{8}$$

where $C_i(t)$ denotes the phase noise of the laser on satellite i at time t and $\tau_{12} = L/c$ denotes the single trip time of flight for light between the two satellites, where c is the speed of light. Note that τ_{12} is different to τ_S. Taking the Fourier transform of this expression we obtain

$$C_{\mathrm{tot}}(f) = C_1(f)(1 - e^{-2\pi i f 2\tau_{12}})\ . \tag{9}$$

As the τ_{12} term refers to a time of flight for light, this will be very small and so using the small angle approximation the absolute value becomes

$$|C_{\mathrm{tot}}(f)| \approx |C_1(f)||4\pi f \tau_{12}|\ . \tag{10}$$

We see that the laser phase noise spectrum scales linearly with τ_{12} or equivalently with L, the distance between the satellites.

Supplementary Note 3 . QUANTUM NOISE SPECTRUM

The are two major sources of optical loss in satellite-to-satellite communications. These are beam diffraction and beam misalignment (pointing error), which ensure that not all of the optical power leaving the first satellite reaches

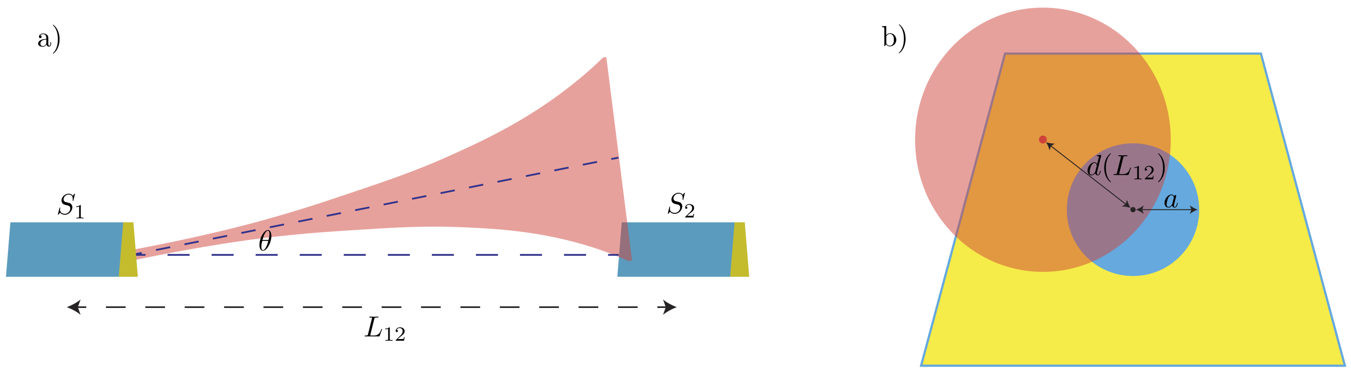

Supplementary Figure 1. **Optical loss mechanism.** a) Through beam diffraction and imperfect beam alignment, most of the power leaving the first satellite does not arrive at the second satellite. b) The receiving aperture on the second satellite has a radius a and the arriving beam is off centre by a distance $d(L)$. As the satellite separation L becomes larger the beam is further off centre for a fixed pointing angle error.

the second satellite. The amount of optical loss is governed by the properties of Gaussian beams. A Gaussian beam expands as it propagates meaning the incoming beam waist at the second satellite is considerably larger than the receiving optics and so most of the light is lost. The amount of optical loss is therefore a function of satellite separation, L. For a Gaussian beam the beam radius at a distance L is

$$w(L) = w_0\sqrt{1 + \left(\frac{L}{z_R}\right)^2} \, , \tag{11}$$

where w_0 is the initial beam waist and

$$z_R = \frac{\pi w_0^2}{\lambda} \, , \tag{12}$$

is the Rayleigh range, where λ is the wavelength of the light. We assume $\lambda = 1064$ nm throughout as this is the wavelength of the laser ranging interferometer in the current mission [2]. It can then be calculated that the power passing through an aperture of radius a, at a distance L is given by

$$P(a, L) = P_0[1 - e^{\frac{-2a^2}{w(L)^2}}] \, , \tag{13}$$

where P_0 is the initial power. This can be modelled as a lossy quantum channel with transmissivity η, such that $P(a, L) = \eta P_0$. Additionally if the beam is not centred (i.e. the centre of the Gaussian beam does not go directly through the centre of the aperture), it is known that this corresponds to a transmissivity of [3]

$$\eta(a, L) = \exp(\frac{-2d(L)^2}{w(L)^2}) \sum_{k=0}^{\infty} \left(\frac{2^k d(L)^{2k}}{w(L)^{2k} k!} \left(1 - \exp\left(-\frac{2a^2}{w(L)^2}\right) \sum_{i=0}^{k} \frac{2^i a^{2i}}{w(L)^{2i} i!} \right) \right) \, , \tag{14}$$

where $d(L)$ is the distance off centre, i.e. the distance between the centre of the Gaussian beam and the centre of the aperture and a is the radius of the aperture (a few cm on GRACE-FO). $d(L)$ is function of satellite separation L because the system will have a certain angular resolution (mRad for GRACE-FO) which gets worse with larger distances. For small offsets, with an angular resolution θ, the distance off centre is

$$d(L) = L\tan(\theta) \approx L\theta \, . \tag{15}$$

This loss mechanism is shown in Supplementary Figure. 1.

Thus, for a given set of satellite parameters, every distance corresponds to a certain optical loss, which can be modelled as a beamsplitter of transmissivity η. We assume the input to the second arm of the beam-splitter is

vacuum as there are very few thermal photons at optical frequencies in space. With this approach, it is simple to examine the impact this channel will have on the mean and covariance matrix of a Gaussian state. After travelling from one satellite to another, a coherent state, initially $\hat{D}(\alpha)\left|0\right\rangle = \left|\alpha\right\rangle$, where $\hat{D}(\alpha)$ is the displacement operator, will have mean and covariance defined by

$$\langle\hat{\alpha}\rangle = \begin{bmatrix} 2\sqrt{\eta}\alpha \\ 0 \end{bmatrix} , \tag{16}$$

$$\Sigma_\alpha = I\ . \tag{17}$$

where I is the 2x2 identity matrix. The average photon number for this coherent state is now $\langle\hat{n}\rangle = \left|\eta\alpha^2\right|$. A displaced squeezed state, $\hat{D}(\alpha)\hat{\mathcal{E}}(r)\left|0\right\rangle$, where $\hat{\mathcal{E}}(r)$ is the squeezing operator and r is the initial squeezing parameter, will suffer a degradation of both the amount and the purity of squeezing as the satellite separation is increased. The effect of the beam-splitter on the covariance matrix of a squeezed beam is given by:

$$\Sigma_{\alpha_r} = \begin{bmatrix} \eta e^{-2r} + (1-\eta) & 0 \\ 0 & \eta e^{2r} + (1-\eta) \end{bmatrix} . \tag{18}$$

This approach offers a simple way to determine how the squeezing level and mean photon number are affected by satellite separation. Hence given a certain input state and satellite separation, the received state is completely determined. From this the quantum noise level is determined. Given an initial power P_0, the power received at the second satellite is $P_{\rm rec} = \eta(L)P_0$. The number of photons received per second is then $\alpha^2 = P_{\rm rec}/(hf)$, where h is Plancks constant and f is the frequency of the light. The minimum allowed standard deviation for measuring phase when quantum noise limited is

$$\triangle\phi = \frac{\sqrt{e^{-2r_{\rm eff}}}}{\sqrt{4\eta\alpha^2}} , \tag{19}$$

where $e^{-2r_{\rm eff}} = \eta e^{-2r} + (1-\eta)$ defines $r_{\rm eff}$, the effective squeezing parameter the second satellite receives. Supplementary Equation. (19) assumes that homodyne detection has been employed to keep the measurement locked to the squeezed quadrature and does not account for optical inefficiencies on board the receiving satellite. The ranging uncertainty is the phase uncertainty multiplied by $\lambda/(2\pi)$, where λ is the wavelength of the light. Quantum noise corresponds to a white noise spectrum and can be converted to an acceleration noise spectrum by multiplying by $(2\pi f)^2$.

Supplementary Note 4 . ALTERNATIVE LASER PHASE NOISE FREE COMBINATIONS

In the main text only one TDI combination was explicitly considered, that with a single laser at the middle satellite, formation α_3^T. This laser light is split and directed towards the two outer satellites, where it is reflected back towards the middle satellite. We now show that many other combinations are possible. For the single laser combinations it is possible to have the laser on either the first or third satellites. This light is sent to the satellites without a laser and reflected back to the satellite with the laser. This arrangement may lead to the interaction of the light with itself as both paths overlap which can lead to further complications. However, this is avoidable, for example, by frequency shifting the light at the distant satellite. With the laser on the first satellite the following combination is laser phase noise free

$$\begin{aligned} &[2\hat{\phi}_{12}(t) - 2\hat{\phi}_{13}(t) - 2\hat{\phi}_{12}(t-2\tau_{12}-2\tau_{23}) + 2\hat{\phi}_{13}(t-2\tau_{12})] \\ =\ &[2\phi_{12}(t) - 2\phi_{13}(t) - (2\phi_{12}(t-2\tau_{12}-2\tau_{23}) - 2\phi_{13}(t-2\tau_{12}))] \\ &+ (QN_{12}(t) - QN_{12}(t-2\tau_{12}-2\tau_{23})) + (QN_{13}(t-2\tau_{12}) - QN_{13}(t)) \\ &+ (AN_{12}(t) - AN_{12}(t-2\tau_{12}-2\tau_{23})) + (AN_{13}(t-2\tau_{12}) - AN_{13}(t))\ . \end{aligned} \tag{20}$$

In this case we can assume the two accelerometer noise terms have the same frequency spectrum but we cannot assume the two quantum noise terms have the same spectrum as they correspond to light which has travelled different distances and hence correspond to different quantum noise levels. For this configuration the signal is transformed as

$$|a_{R,\mathrm{TDI}}(f)| = \frac{8\pi f GM}{v_0^2}\left|K_0\left(\frac{2\pi f}{f_h}\right)\right|\Bigg|\Bigg(- e^{-2\pi \mathrm{i} f\tau_S} + e^{-2\pi \mathrm{i} f(2\tau_S)} - e^{-2\pi \mathrm{i} f(4\tau)} + e^{-2\pi \mathrm{i} f(\tau_S+4\tau)} + e^{-2\pi \mathrm{i} f(2\tau)} - e^{-2\pi \mathrm{i} f(2\tau_S+2\tau)}\Bigg)\Bigg| , \tag{21}$$

where $\tau = \tau_{12} = \tau_{23}$ is the single trip time of flight for light between the two satellites, assuming the satellite separations are equal. Although it is not obvious, in the frequency domain, this is very similar to the combination with the laser at the middle satellite. We consider the accelerometer noise as two separate contributions each with the same noise spectrum but with a different delay, which changes the total accelerometer noise spectrum to $\left|AN_{13}(f)(1 - e^{-2\pi \mathrm{i} f2\tau}) - AN_{12}(f)(1 - e^{-2\pi \mathrm{i} f4\tau})\right|$. Similarly, the quantum noise spectrum will transform to $\left|QN_{13}(f)(1 - e^{-2\pi \mathrm{i} f2\tau}) - QN_{12}(f)(1 - e^{-2\pi \mathrm{i} f4\tau})\right|$. Essentially the same analysis holds for the combination which has the laser on the third satellite. It may be possible to simultaneously operate all three single laser configurations at different wavelengths. This would provide extra information and still allow the removal of ionospheric effects [4].

We now consider alternate, multi-laser schemes which have all been explored for LISA [5]. We examine a 3 laser, 6 measurement configuration which includes acceleration and quantum noise. The 6 measured phases are

$$\hat{\phi}_{12} = \phi_{12} + C_2(t - \tau_{12}) - C_1(t) + AN_{12}(t) + QN_{12}(t) \tag{22}$$

$$\hat{\phi}_{13} = \phi_{13} + C_3(t - \tau_{13}) - C_1(t) + AN_{13}(t) + QN_{13}(t) \tag{23}$$

$$\hat{\phi}_{21} = \phi_{12} + C_1(t - \tau_{12}) - C_2(t) + AN_{21}(t) + QN_{21}(t) \tag{24}$$

$$\hat{\phi}_{23} = \phi_{23} + C_3(t - \tau_{23}) - C_2(t) + AN_{23}(t) + QN_{23}(t) \tag{25}$$

$$\hat{\phi}_{31} = \phi_{13} + C_1(t - \tau_{13}) - C_3(t) + AN_{31}(t) + QN_{31}(t) \tag{26}$$

$$\hat{\phi}_{32} = \phi_{23} + C_2(t - \tau_{23}) - C_3(t) + AN_{32}(t) + QN_{32}(t) \tag{27}$$

where we have assumed the time of flights are symmetric, i.e. $\tau_{12} = \tau_{21}$. From these 6 measurements the following 3 laser phase noise free combinations can be constructed.

$$\alpha(t) = \hat{\phi}_{13}(t) - \hat{\phi}_{12}(t) + \hat{\phi}_{32}(t - \tau_{13}) - \hat{\phi}_{23}(t - \tau_{12}) + \hat{\phi}_{21}(t - \tau_{23} - \tau_{13}) - \hat{\phi}_{31}(t - \tau_{23} - \tau_{12}) \tag{28}$$

$$\beta(t) = \hat{\phi}_{21}(t) - \hat{\phi}_{23}(t) + \hat{\phi}_{13}(t - \tau_{12}) - \hat{\phi}_{31}(t - \tau_{23}) + \hat{\phi}_{32}(t - \tau_{13} - \tau_{12}) - \hat{\phi}_{12}(t - \tau_{13} - \tau_{23}) \tag{29}$$

$$\gamma(t) = \hat{\phi}_{32}(t) - \hat{\phi}_{31}(t) + \hat{\phi}_{21}(t - \tau_{23}) - \hat{\phi}_{12}(t - \tau_{13}) + \hat{\phi}_{13}(t - \tau_{23} - \tau_{12}) - \hat{\phi}_{23}(t - \tau_{13} - \tau_{12}) \tag{30}$$

These schemes can be compared to the single laser TDI schemes by examining how the signal and various noise sources are transformed by these combinations. In each combination the signal remaining is

$$\alpha_{sig}(t) = \phi_{23}(t) - \phi_{23}(t - \tau_{12}) + \phi_{12}(t - \tau_{12} - 2\tau_{23}) - \phi_{12}(t - \tau_{12} - \tau_{23}), \tag{31}$$

$$\beta_{sig}(t) \approx \; \phi_{12}(t) - \phi_{23}(t) - \phi_{12}(t - \tau_{12} - 2\tau_{23}) + \phi_{23}(t - \tau_{12} - \tau_{23}), \tag{32}$$

$$\gamma_{sig}(t) = \; - \phi_{12}(t) + \phi_{12}(t - \tau_{23}) + \phi_{23}(t - \tau_{12} - \tau_{23}) - \phi_{23}(t - 2\tau_{12} - \tau_{23}), \tag{33}$$

where we have used the fact that $\tau_{13} = \tau_{12} + \tau_{23}$ and $\phi_{13} = \phi_{12} + \phi_{23}$. It is possible to convert to the frequency domain, where we see that these combinations result in a greatly reduced signal. We can also note that in each combination no measurement is used twice, and so each combination contains the quantum noise 6 times. Thus, the quantum noise is not reduced in the same way that the signal and accelerometer noise are. These schemes do not perform as well as the single laser schemes.

Supplementary Note 5 . GRAVITATIONAL SIGNAL AFTER TDI

As discussed in the main text, by switching to a three satellite configuration and using TDI, the laser phase noise can be completely removed from the measurement. With a single laser on the middle satellite, sent to the outer satellites and back, formation α_3^T, the following combination of the measured phases completely removes laser phase noise.

$$2([\hat{\phi}^{\mathrm{g}}_{21}(t) - \hat{\phi}^{\mathrm{g}}_{23}(t)] - [\hat{\phi}^{\mathrm{g}}_{21}(t - 2\tau_{23}) - \hat{\phi}^{\mathrm{g}}_{23}(t - 2\tau_{21})]) \;, \tag{34}$$

where $\hat{\phi}^{\mathrm{g}}_{ij}(t)$ denotes the measured phase shift at time t using light received at satellite i from satellite j. Converting the gravitational phase shift to the accelerations of the different satellites (e.g. $\hat{\phi}^{\mathrm{g}}_{21}(t) \to a_{\mathrm{g},1}(t) - a_{\mathrm{g},2}(t)$) gives the ranging signal as

$$\begin{aligned} a_{R,\mathrm{TDI}}(t) = 2([&a_{\mathrm{g},1}(t) - a_{\mathrm{g},2}(t) - (a_{\mathrm{g},2}(t) - a_{\mathrm{g},3}(t))] \\ &-[a_{\mathrm{g},1}(t - 2\tau_{23}) - a_{\mathrm{g},2}(t - 2\tau_{23}) - (a_{\mathrm{g},2}(t - 2\tau_{21}) - a_{\mathrm{g},3}(t - 2\tau_{21}))]) \;, \end{aligned} \tag{35}$$

where $a_{\mathrm{g},i}(t)$ is the range acceleration of the ith satellite at time t. We will assume that the satellites are all separated by the same distance, so that $\tau_L = \tau_{21} = \tau_{23}$, where the subscript L denotes that we are referring to a light time of flight, and $a_{\mathrm{g},3}(t) = a_{\mathrm{g},2}(t - \tau_S) = a_{\mathrm{g},1}(t - 2\tau_S)$. Converting to the frequency domain then gives

$$\begin{aligned} a_{R,\mathrm{TDI}}(f) &= 2a(f)[(1 - e^{-2\pi \mathrm{i} f\tau_S} - (e^{-2\pi \mathrm{i} f\tau_S} - e^{-2\pi \mathrm{i} f 2\tau_S})) \\ &\quad - (e^{-2\pi \mathrm{i} f 2\tau_L} - e^{-2\pi \mathrm{i} f(\tau_S + 2\tau_L)} - (e^{-2\pi \mathrm{i} f(\tau_S + 2\tau_L)} - e^{-2\pi \mathrm{i} f(2\tau_S + 2\tau_L)}))] \\ &= 2a(f)(1 - e^{-2\pi \mathrm{i} f\tau_S} - (e^{-2\pi \mathrm{i} f\tau_S} - e^{-2\pi \mathrm{i} f 2\tau_S})) \times (1 - e^{-2\pi \mathrm{i} f 2\tau_L}) \\ &= 2a(f)(1 - e^{-2\pi \mathrm{i} f\tau_S})^2(1 - e^{-2\pi \mathrm{i} f 2\tau_L}) \;. \end{aligned} \tag{36}$$

Taking the absolute value of this gives the expression in the main text, Eq. (10). The acceleration range after TDI is proportional to $|\sin(\pi f/f_c)|$, where $f_c = c/(2L)$. This introduces nulls in the signal at certain frequencies which are not present in the original ranging signal. After rescaling by a factor $1/(2\sqrt{2}|\sin(\pi f/f_c)|)$, so that quantum noise is unaffected by TDI, the signal is affected by a $\sqrt{2}|\sin(2\pi f/f_L)|$ term compared to the signal without TDI. The main effect of this is to reduce the signal at low frequencies. Close to the frequency of interest TDI does not affect the signal significantly.

Supplementary Note 6 . TDI WITH UNEQUAL SATELLITE SEPARATIONS

For completeness we now consider the first TDI combination presented, i.e. formation α_3^T, in the situation where the satellite separations are not equal. The acceleration combination which is laser phase noise free is

$$\begin{aligned} a_{\rm TDI} &= a_{g,12}(t) - a_{g,23}(t) - (a_{g,12}(t-2\tau_{23}) - a_{g,23}(t-2\tau_{12})) \\ &= a_{g,1}(t) - a_{g,2}(t) - a_{g,2}(t) + a_{g,3}(t) - \\ &\quad (a_{g,1}(t-2\tau_{23}) - a_{g,2}(t-2\tau_{23}) - a_{g,2}(t-2\tau_{12}) + a_{g,3}(t-2\tau_{12})) \\ &= a_{g,1}(t) - 2a_{g,1}(t-\tau_{S12}) + a_{g,1}(t-\tau_{S12}-\tau_{S23}) - \\ &\quad (a_{g,1}(t-2\tau_{23}) - a_{g,1}(t-\tau_{S12}-2\tau_{23}) - a_{g,1}(t-\tau_{S12}-2\tau_{12}) + a_{g,1}(t-\tau_{S12}-\tau_{S23}-2\tau_{12}))\ , \end{aligned} \tag{37}$$

where we use the fact that the accelerations of all the satellites are equal but delayed in time and τ_{ij} refers to the light time of travel and τ_{Sij} refers to the satellite time of travel. Taking the Fourier transform we find

$$\begin{aligned} a_2(f) = \ & a(f)[(1-e^{-2\pi i f(2\tau_{23})}) + e^{-2\pi i f(\tau_{S12})}(-2 - e^{-2\pi i f(2\tau_{23})} + e^{-2\pi i f(2\tau_{12})}) \\ & + e^{-2\pi i f(\tau_{S12}+\tau_{S23})}(1-e^{-2\pi i f(2\tau_{12})})]\ . \end{aligned} \tag{38}$$

where $a(f)$ is the acceleration frequency spectrum of a single satellite, as given in Supplementary Equation. (5) and the subscript 2 refers to the fact that this combination has a laser on-board the middle satellite (satellite 2). When the satellite separations are the same, $\tau_{S12} = \tau_{S23} = \tau_S$ and $\tau_{12} = \tau_{23} = \tau$, then this reduces to $a(f)(1-e^{-2\pi i f(2\tau)})(1-e^{-2\pi i f(\tau_S)})^2$, as expected. Similarly, if the laser is at satellite 1 initially, the signal after TDI becomes

$$\begin{aligned} a_1(f) = \ & a(f)[e^{-2\pi i f(\tau_{S12}+\tau_{S23})} - e^{-2\pi i f(\tau_{S12})} - e^{-2\pi i f(2\tau_{12}+2\tau_{23})} + e^{-2\pi i f(\tau_{S12}+2\tau_{23}+2\tau_{12})} \\ & + e^{-2\pi i f(2\tau_{12})} - e^{-2\pi i f(2\tau_{12}+\tau_{S12}+\tau_{S23})}]\ . \end{aligned} \tag{39}$$

With the laser at satellite 3 the signal after TDI becomes

$$\begin{aligned} a_3(f) = \ & a(f)[e^{-2\pi i f\tau_{S12}} - 1 - e^{-2\pi i f(2\tau_{12}+2\tau_{23}+\tau_{S12})} + e^{-2\pi i f(2\tau_{23})} + e^{-2\pi i f(\tau_{S12}+\tau_{S23}+2\tau_{23}+2\tau_{12})} \\ & - e^{-2\pi i f(2\tau_{23}+\tau_{S12}+\tau_{S23})}]\ . \end{aligned} \tag{40}$$

The accelerometer noise after TDI, with the laser at the middle satellite, transforms as

$$AN_{\rm TDI,2}(f) \rightarrow AN_{21}(f)(1-e^{-2\pi i f 2\tau_{23}}) + AN_{23}(f)(1-e^{-2\pi i f 2(\tau_{12})})\ . \tag{41}$$

Similarly, if the laser is at satellite 1 or 3 the accelerometer noise transforms as

$$AN_{\rm TDI,1}(f) \rightarrow AN_{21}(f)(1-e^{-2\pi i f 2(\tau_{12}+\tau_{23})}) + AN_{13}(f)(1-e^{-2\pi i f 2(\tau_{12})})\ , \tag{42}$$

or

$$AN_{\rm TDI,3}(f) \rightarrow AN_{23}(f)(1-e^{-2\pi i f 2(\tau_{12}+\tau_{23})}) + AN_{13}(f)(1-e^{-2\pi i f 2(\tau_{23})})\ , \tag{43}$$

respectively. For the accelerometer instrument noise, the various $AN_{i,j}$ terms above will be statistically similar. However, for the quantum noise as the two arms are different lengths the quantum noise levels in the two measurements will be different, hence the corresponding $QN_{i,j}$ terms will not be statistically similar. This allows the minimum detectable mass to be examined as a function of both satellite separations, as shown in Supplementary Figure. 2. However, this does not reveal any interesting new optimal regimes for satellite geodesy. As expected, we see that the minimum detectable mass is symmetric in both arm lengths.

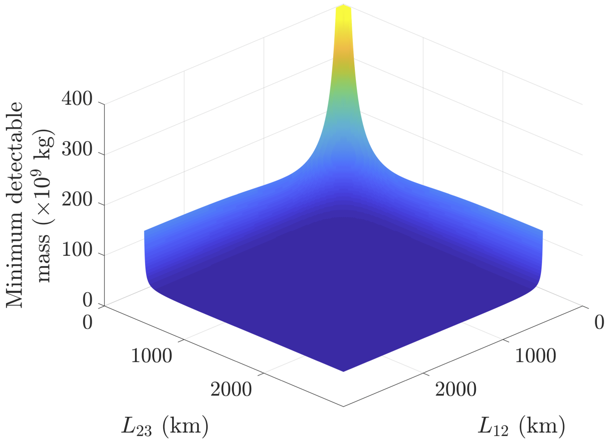

Supplementary Figure 2. **Minimum detectable mass using TDI with different satellite separations.** The minimum detectable mass is shown as a function of the distance between satellites 1 and 2, L_{12} and between satellites 2 and 3, L_{23}, when using time delay interferometry. Figure is shown for satellites at an orbital height of 500 km with an accelerometer noise of $a_0 = 1 \times 10^{-12}$ m s$^{-2}\sqrt{\text{Hz}}^{-1}$. Quantum noise is negligible at this level of accelerometer noise.

Supplementary Note 7 . EXPERIMENTAL ASPECT OF TDI

In principle TDI allows for the perfect removal of laser phase noise. This relies on time-delaying the data series by a time corresponding exactly to the time of flight of light travelling between the two satellites. However, in reality the satellite arm length will not be known perfectly, rather it will be known to within some uncertainty, $\hat{\tau}_{ij} = \tau_{ij} + \delta_j$, where $\hat{\tau}_{ij}$ is the estimated time of flight and δ_j is a random variable which represents the error in this estimate [6]. Substituting the estimated time of flight into the TDI combinations shows that without perfect arm length knowledge there will be some left-over laser phase noise. For formation α_3^T with a single laser on the middle satellite, the left-over laser phase noise is

$$\begin{aligned} \text{LPN}_{\text{left-over}} = {} & C_2(t - 2\tau_{21}) - C_2(t - 2\tau_{21} - 2\delta_1) - C_2(t - 2\tau_{23}) + C_2(t - 2\tau_{23} - 2\delta_3) \\ & + C_2(t - 2\tau_{21} - 2\tau_{23} - 2\delta_3) - C_2(t - 2\tau_{21} - 2\tau_{23} - 2\delta_1) \ . \end{aligned} \tag{44}$$

In order to calculate the remaining laser phase noise after TDI we expand about $\delta_i = 0$ to first order and assume the uncertainty in both arm lengths is the same, $\delta_1 = \delta_3 = \delta$. In the time domain this gives

$$\text{LPN}_{\text{left-over}} = \ 2\delta(\dot{C}_2(t - 2\tau_{21}) - \dot{C}_2(t - 2\tau_{23})) \ . \tag{45}$$

Converting to the frequency domain we obtain the new laser phase noise spectrum.

$$\sqrt{S_{\text{LPN}_{\text{left-over}}}(f)} \approx 8\pi\delta f \sqrt{S_{\text{LPN}}} \ . \tag{46}$$

This is the approach we use for the simulations in the main text. However, as an alternative approach which may be more intuitive, we can note that the model being used for laser phase noise is linear in satellite separation. Assuming this holds for small distances, we can simply look at the total "distance" which remains in the expression for left-over laser phase noise, i.e. $4\delta_1 + 4\delta_3$. A pessimistic estimate for the left-over laser phase noise amplitude spectrum is then

$$\sqrt{S_{\text{LPN}_{\text{left-over}}}(f)} = \frac{(2\pi f)^2 x_T 8\delta_d}{\sqrt{f}} \ , \tag{47}$$

where we assume that $\delta_1 \approx \delta_3$ and $\delta_d = c\delta_1$ is a distance as opposed to a time. The requirement to be quantum noise limited when using this TDI combination is that the power spectrum of the left-over laser phase noise is smaller

than that of the quantum noise spectrum, $\left|\sqrt{S_{\mathrm{LPN}_{\text{left-over}}}(f)}\right| \leq \sqrt{S_{\mathrm{QN,TDI}}(f)}$. There is a similar requirement on the accelerometer noise, in order for the left-over laser phase noise to be unimportant. This is more relevant to the present mission as accelerometer noise is larger than quantum noise. This places a requirement on how accurately the arm lengths must be known for TDI to be beneficial. With GPS positioning accuracy on the order of 5 mm [7, 8], this requirement is easily surpassed. Millimetre level positioning is sufficiently accurate for TDI not to be a limiting factor even for $a_0 = 1 \times 10^{-15}$ m s$^{-2}\sqrt{\mathrm{Hz}}^{-1}$. Thus, TDI offers a practical and attainable method of improving the sensitivity of geodesy missions.

Supplementary Note 8 . NOISE SOURCES AFTER TDI

When taking the above TDI combination several noise terms are combined in the final expression. The different noise terms combine in different ways depending on whether they are correlated or not. We consider quantum noise first. Adding two uncorrelated quantum noise terms, is adding two series of time data with a certain variance, σ_1^2 and σ_2^2. The total variance is $\sigma_{\mathrm{T}}^2 = \sigma_1^2 + \sigma_2^2$. This has power spectral density proportional to σ_{T}^2 and so amplitude spectral density proportional to $\sigma_{\mathrm{T}} = \sqrt{\sigma_1^2 + \sigma_2^2}$. Assuming the two quantum noise spectra being combined are statistically similar (same variance) we see that $\sigma_{\mathrm{T}} = \sqrt{2}\sigma_1$, i.e. amplitude spectra get scaled by a factor $\sqrt{2}$. Adding N quantum noise terms together will scale the total quantum noise amplitude spectrum by a factor of $\sqrt{N}$, compared to each individual spectrum (assuming all the spectra are statistically similar).

This is true for quantum noise which has a white noise spectrum. However, the above argument is easily extended to any shape of frequency spectrum. Any noise spectrum, $A(f)$, has a shape which depends on the frequency. This function determines the magnitude of the noise at that frequency. In order to obtain a noisy frequency spectrum the magnitude of the spectrum at each frequency can be multiplied by a normally distributed random number with mean 0 and variance 1, $\mathcal{N}(0,1)$, to change its magnitude and multiplied by a random complex number, $e^{\mathrm{i}\theta}$, where θ is distributed uniformly in the region 0 to 2π, to change its phase. We then consider adding j of these similar noise spectra together. At a given frequency, f, we can model the total frequency spectrum as

$$|A(f)| \sum_j e^{\mathrm{i}\theta_j} \mathcal{N}_j(0,1) \; . \tag{48}$$

The absolute value of this spectrum then indicates how much noise we can expect at a given frequency. As the spectral shape $A(f)$ is outside the sum, at any given frequency the expected value of this is simply $\sqrt{j}$ times larger than a single noise spectra. This is true at all frequencies and for any spectral shape.

We can now use this to calculate how the quantum noise or accelerometer noise spectrum is affected by TDI. From Eq. (9) in the main text, the quantum noise in the time domain after taking the TDI combination is

$$QN_{\mathrm{TDI}}(t) = (QN_{21}(t) - QN_{21}(t - 2\tau_{23})) - (QN_{23}(t) - QN_{23}(t - 2\tau_{21})) \; , \tag{49}$$

where $QN_{ij}(t)$ ($AN_{ij}(t)$) represent the quantum noise (accelerometer noise) for a measurement using light received at satellite i from satellite j. Converting to the frequency domain gives

$$QN_{\mathrm{TDI}}(f) = QN_{21}(f)(1 - e^{-2\pi \mathrm{i} f(2\tau_{23})}) + QN_{23}(f)(1 - e^{-2\pi \mathrm{i} f(2\tau_{21})}) \; , \tag{50}$$

which, assuming that the two quantum noise spectra are statistically equivalent and that the satellites separations are initially equal, gives

$$|QN_{\mathrm{TDI}}(f)| = 2\sqrt{2} QN(f) \left| \sin(\frac{2\pi f}{f_c}) \right| \; . \tag{51}$$

The accelerometer noise is transformed in the same way. The remaining laser phase noise is in theory perfectly cancelled. However, with experimental imperfections, this is not the case, as discussed in Supplementary Note 7 .

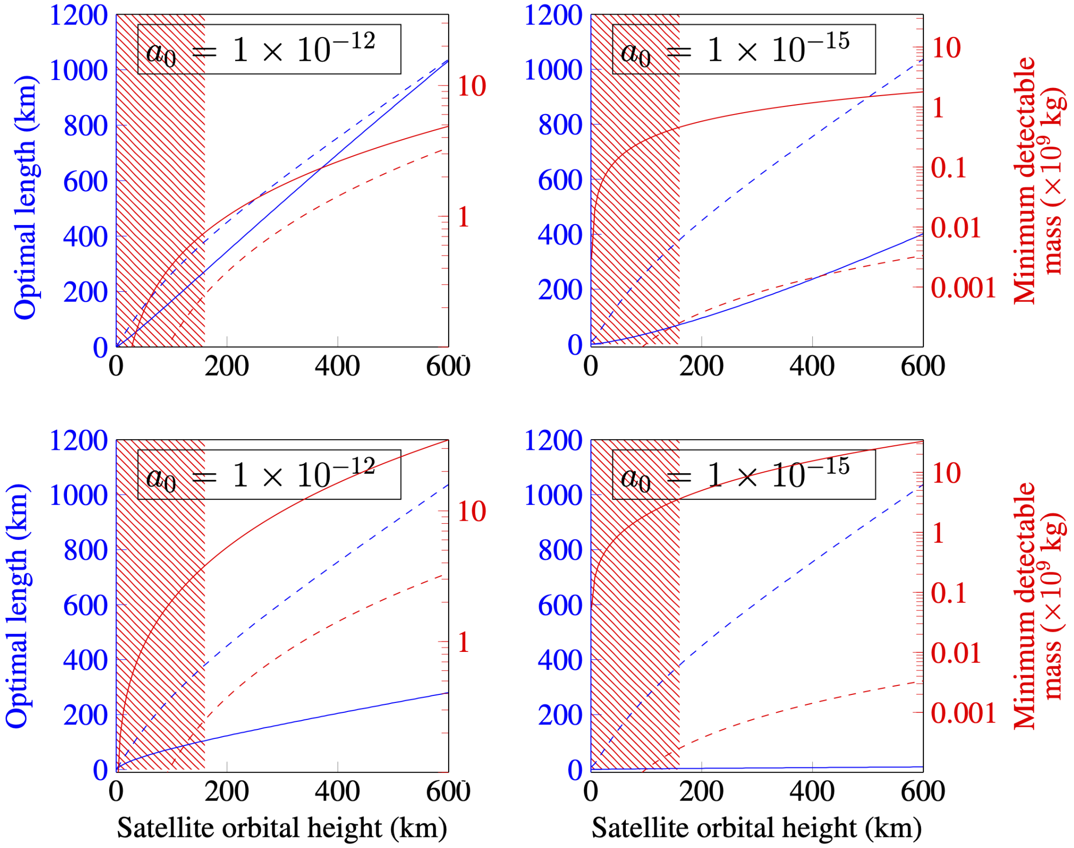

Supplementary Figure 3. **Minimum detectable mass and corresponding optimal satellite separation for different satellite orbital heights.** The data is shown for two different accelerometer instrument noise levels, $a_0 = 1 \times 10^{-12}$ m s$^{-2}\sqrt{\mathrm{Hz}}^{-1}$ and $a_0 = 1 \times 10^{-15}$ m s$^{-2}\sqrt{\mathrm{Hz}}^{-1}$ both with (dashed lines) and without (solid lines) TDI. The hashed red region represents satellite orbital heights below low Earth orbit (160 km) and so this region is not feasible. The top and bottom rows show the same data but using two different initial laser phase noise spectra, corresponding to the actual laser phase noise performance ($x_T = 1 \times 10^{-15}$, top row) and the laser phase noise requirement ($x_c = 8 \times 10^{-9}$ m $\sqrt{\mathrm{Hz}}^{-1}$, bottom row) respectively.

Supplementary Note 9 . MINIMUM DETECTABLE MASS

The minimum detectable mass quantifies the useful region of the frequency spectrum of the gravitational signal and all the noise sources. When using a matched filter, which is optimal if the shape of the signal is known, the SNR of the gravitational ranging system for detecting a point mass M is [9]

$$\rho = 4 \int_0^\infty \frac{|a_\mathrm{R}(f)|^2}{S_\mathrm{T}(f)} df \ , \tag{52}$$

where $a_\mathrm{R}(f)$ is the gravitational signal and $S_\mathrm{T}(f)$ is the combined power spectrum of all the different noise sources in the system. Hence the SNR per unit mass is

$$\rho' = 4 \int_0^\infty \frac{|a_\mathrm{R}(f)/M|^2}{S_\mathrm{T}(f)} df \ . \tag{53}$$

In the main text the minimum detectable mass was defined in Eq. (15) as

$$M_\mathrm{min} = \frac{3}{\sqrt{4 \int_0^\infty \frac{|a_\mathrm{R}(f)/M|^2}{S_\mathrm{T}(f)} df}} \ . \tag{54}$$

It is now clear that the minimum detectable mass is simply the smallest mass which gives a SNR of at least 3. Intuitively this concept represents the smallest possible mass which the satellite system can detect. This concept allows the optimal satellite separation (that which minimises the minimum detectable mass) to be determined for a given orbital height. In the main text this was presented for satellites at an orbital height of 500 km. This orbital height was chosen as it is the orbital height of the current GRACE mission. We now present the optimal satellite separation, and the corresponding minimum detectable mass, as a function of satellite orbital height in Supplementary Figure. 3. The advantage of improving the accelerometer instrument noise by 3 orders of magnitude is very marginal without TDI. This is because without TDI, laser phase noise remains the dominant noise source and so improving the accelerometer does not help.

Supplementary Note 10 . ALTERNATIVE ACCELEROMETER NOISE FREE COMBINATIONS

In the main text we briefly mentioned how accelerometer noise free combinations can be obtained from TDI combinations using three satellites of different masses (M_1, M_2 and M_3), formation $\alpha^T_{3,DM}$ in the main text. This formation works based on the assumption that the non-gravitational accelerations experienced by the satellites consists of a stationary, $a^{\rm ng}_{\rm s}$, and a non-stationary, $a^{\rm ng}_{\rm ns}$, component. All satellites experience the same stationary component and the closer the satellites are to each other the more similar the non-stationary components they experience will be. We can formalise this as $a^{\rm ng}_2(t) = a^{\rm ng}_1(t-\tau_s) + \delta_{a2}(\mathrm{t})$, where $a^{\rm ng}_i$ is the non-gravitational acceleration experienced at satellite i, δ_{ai} is the difference between the non-gravitational acceleration of the first satellite and the non-gravitational acceleration of satellite i at the same position and τ_s is the time it takes the second satellite to reach the position of the first one. Calling L_i, the single laser TDI combination with a laser at the ith satellite (i.e. $L_2(t)$ is given in Eq. (9) in the main text), the following combinations of TDI combinations cancel the stationary component of the accelerometer noise

$$\begin{aligned}\mathrm{Com}_1 = {} & (L_1(t) - L_1(t-2\tau_{23}) + \frac{M_1}{M_3}L_1(t-2\tau_S) - \frac{M_1}{M_3}L_1(t-2\tau_{21}-2\tau_S) - 2\frac{M_1}{M_2}L_1(t-\tau_S) \\ & + \frac{M_1}{M_2}L_1(t-2\tau_{21}-\tau_S) + \frac{M_1}{M_2}L_1(t-2\tau_{23}-\tau_S)) - [L_2(t-2\tau_{21}) - L_2(t-2\tau_{21}-2\tau_{23}) \\ & + \frac{M_1}{M_3}L_2(t-2\tau_S) - \frac{M_1}{M_3}L_2(t-2\tau_{21}-2\tau_S) - \frac{M_1}{M_2}L_2(t-\tau_S) + \frac{M_1}{M_2}\ L_2(t-2\tau_{21}-2\tau_{23}-\tau_S)]\ .\end{aligned} \tag{55}$$

$$\begin{aligned}\mathrm{Com}_2 = {} & (-L_1(t) + L_1(t-2\tau_{23}) - \frac{M_1}{M_3}L_1(t-2\tau_S-2\tau_{23}) + \frac{M_1}{M_3}L_1(t-2\tau_{21}-2\tau_S-2\tau_{23}) + \frac{M_1}{M_2}L_1(t-\tau_S) \\ & - \frac{M_1}{M_2}L_1(t-2\tau_{21}-2\tau_{23}-\tau_S)) + \frac{M_1}{M_3}L_3(t-2\tau_S) - [L_3(t-2\tau_{21}) - L_3(t-2\tau_{21}-2\tau_{23}) \\ & - \frac{M_1}{M_3}L_3(t-2\tau_{21}-2\tau_S) - \frac{M_1}{M_2}L_3(t-\tau_S) + \frac{M_1}{M_2}L_3(t-2\tau_21-2\tau_23-\tau_S)]\ ,\end{aligned} \tag{56}$$

$$\begin{aligned}\mathrm{Com}_3 = {} & (L_3(t) - L_3(t-2\tau_{23}) + \frac{M_1}{M_3}L_3(t-2\tau_S) - \frac{M_1}{M_3}L_3(t-2\tau_{21}-2\tau_S) - 2\frac{M_1}{M_2}L_3(t-\tau_S) \\ & + \frac{M_1}{M_2}L_3(t-2\tau_{21}-\tau_S) + \frac{M_1}{M_2}L_3(t-2\tau_{23}-\tau_S)) - [-L_2(t) + L_2(t-2\tau_{23}) - \frac{M_1}{M_3}L_2(t-2\tau_S-2\tau_{23}) \\ & + \frac{M_1}{M_3}L_2(t-2\tau_{21}-2\tau_S-2\tau_{23}) + \frac{M_1}{M_2}L_2(t-\tau_S) - \frac{M_1}{M_2}L_2(t-2\tau_{21}-2\tau_{23}-\tau_S)]\ .\end{aligned} \tag{57}$$

As the laser phase noise is already removed in the TDI combinations used here, L_1, L_2 and L_3, these terms only have quantum noise and non-stationary non-gravitational accelerations remaining. However, there are many issues with these combinations; engineering difficulties, greatly reduced signal and the remaining noise sources. As such these schemes appear to have limited practical value.

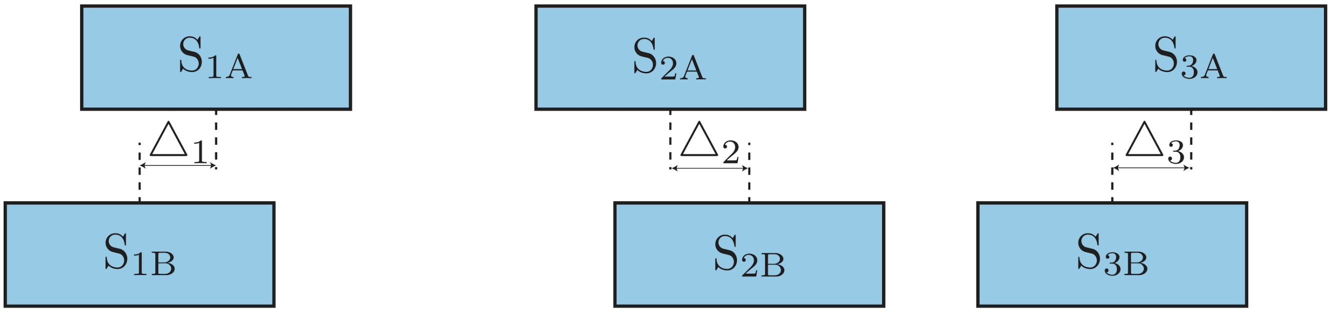

Supplementary Figure 4. **Satellite drift for the six satellite formation presented in the main text, formation $\alpha^T_{6,DM}$.** The three satellite-pairs drift by distances $\triangle_1$, $\triangle_2$ and $\triangle_3$, which are all of a similar order of magnitude. The smaller the satellite-pair drift can be made the better the performance of this scheme. The inter-satellite separation used for the ranging measurement is on the order of hundreds of kilometres.

Supplementary Note 11 . REMAINING NOISES WITH SIX SATELLITE FORMATION FLYING

The differential mass 6 satellite formation presented in the main text, formation $\alpha^T_{6,DM}$, exactly cancels the non-gravitational accelerations and the laser phase noise in an ideal implementation. However, in reality each satellite pair will drift apart. If the three satellite pairs drift apart by distances $\triangle_1$, $\triangle_2$ and $\triangle_3$, as shown in Supplementary Figure. 4, the remaining laser phase noise is

$$\begin{aligned}\mathrm{LPN}_{\text{left-over}} = {} & 2(C_2(t-2\tau_{21}+2\triangle_{t1}) - C_2(t-2\tau_{21}+2\triangle_{t2}) - C_2(t-2\tau_{23}+2\triangle_{t3}) \\ & + C_2(t-2\tau_{23}+2\triangle_{t2}) - C_2(t-2\tau_{21}-2\tau_{23}+2\triangle_{t1}) + C_2(t-2\tau_{21}-2\tau_{23}+2\triangle_{t3}))\ ,\end{aligned} \tag{58}$$

where $\triangle_{ti} = \triangle_i/v_0$, is the time corresponding to each satellite drift. Following the same logic as used in Supplementary Note 7 , this leads to the following expression for the remaining laser phase noise, assuming all the satellites drift a similar distance, $\triangle_i$=$\triangle$,

$$\sqrt{S_{\mathrm{LPN}}(f)} = \frac{(2\pi f)^2 x_T 12\triangle}{\sqrt{f}}\ . \tag{59}$$

This however, assumes that even if the satellites drift apart we take the same naive TDI combination. Alternatively, we can take a different TDI combination which is able to almost completely remove laser phase noise. We form the two quantities $\phi_{T21}(t)$ and $\phi_{T23}(t)$ as described in the main text ($2\phi_{T2i}(t) = 4\hat{\phi}_{B2i}(t) - 2\hat{\phi}_{A2i}(t)$). The laser phase noise in these quantities is

$$C_{T21}(t) = 2C(t-2\tau_{21}+\triangle_{t1}) - C(t-2\tau_{21}) - 2C(t+\triangle_{t2}) + C(t)\ , \tag{60}$$

and

$$C_{T23}(t) = 2C(t-2\tau_{23}+\triangle_{t3}) - C(t-2\tau_{23}) - 2C(t+\triangle_{t2}) + C(t)\ . \tag{61}$$

The following combination is laser phase noise free

$$\begin{aligned}\phi_{6,\mathrm{T}}(t) = {} & \phi_{T21}(t) - \phi_{T23}(t) - [\phi_{T21}(t-2\tau_{23}) - \phi_{T23}(t-2\tau_{21})] \\ & + 2[\phi_{T21}(t-2\tau_{23}+\triangle_{t3}) - \phi_{23}(t-2\tau_{21}+\triangle_{t1})] - 2[\phi_{T21}(t+\triangle_{t2}) - \phi_{T23}(t+\triangle_{t2})]\ ,\end{aligned} \tag{62}$$

where we use the term $\phi_{6,\mathrm{T}}$ to reflect that this corresponds to formation $\alpha^T_{6,DM}$. As in Supplementary Note 7 , including errors in our knowledge of the satellite separations allows an approximate model for the laser phase noise

remaining after taking this combination to be calculated. Doing this indicates that the remaining laser phase noise in this six satellite configuration will be no more than an order of magnitude larger than the laser phase noise left over in our three satellite configuration. Remarkably, even though there are many more terms in this TDI combination than the more simple TDI configuration initially chosen, the frequency domain signals of the two combinations are very similar. In the frequency domain this signal has the following form

$$\begin{aligned} a_{6,\mathrm{TDI}}(f) = a_R(f)[1 - e^{-2\pi i f \tau_S} - [e^{-2\pi i f 2\tau_L} - e^{-2\pi i f(2\tau_L+\tau_S)}] \\ + 2[e^{-2\pi i f(2\tau_L - \triangle_{t3})} - e^{-2\pi i f(2\tau_L - \triangle_{t1} + \tau_S)}] - 2[e^{-2\pi i f(-\triangle_{t2})} - e^{-2\pi i f(\tau_S - \triangle_{t2})}]] \,, \end{aligned} \tag{63}$$

where $a_R(f)$ is the range acceleration signal given by Supplementary Equation. (7) and as before, τ_L and τ_S are the single trip time of flight for light and the satellites respectively. When $\triangle_1 = \triangle_2 = \triangle_3 = 0$, this reduces to the normal TDI signal. When this TDI combination is taken laser phase noise is almost completely removed. However, the other noise sources remain in the measurement. When the individual measurements are combined, $\phi_{Ti,j} = 2\phi_{Bi,j} - \phi_{Ai,j}$, the quantum noise in the $\phi_{Bi,j}$ term is doubled. This combined with the noises in $\phi_{Ai,j}$ (which we assume to be statistically equivalent) means the quantum noise in the $\phi_{Ti,j}$ term is a factor of $\sqrt{5}$ larger than in the $\phi_{A(B)i,j}$ terms. In the final TDI expression there are then two independent quantum noise terms (one each from $\phi_{T21}(t)$ and $\phi_{T23}(t)$). For this TDI combination the quantum noise is transformed as

$$\begin{aligned} QN(f) \to QN'_{21}(f)[1 - e^{-2\pi i f 2\tau_L} + 2e^{-2\pi i f(2\tau_L - \triangle_{t3})} - 2e^{2\pi i f \triangle_{t2}}] \\ + QN'_{23}(f)[-1 + e^{-2\pi i f 2\tau_L} - 2e^{-2\pi i f(2\tau_L - \triangle_{t1})} + 2e^{2\pi i f \triangle_{t2}}] \,, \end{aligned} \tag{64}$$

where we have assumed the satellite separations are initially equal and $QN'_{ij}(f)$ represents the quantum noise spectrum in the $\phi_{Ti,j}$ terms, i.e. the normal quantum noise scaled by $\sqrt{5}$. There is some accelerometer noise left in each $\phi_{Ti,j}$ term, owing to imperfect satellite flying, which transforms in a similar way. The combination of all of these left-over noises gives the total noise spectrum for this formation.

Supplementary Note 12 . ESTIMATING PHASE WHEN QUANTUM NOISE LIMITED

Naively one might imagine that the problem of computing the ultimate precision in satellite geodesy is a typical phase estimation problem [10]. Upon delving deeper into the problem it becomes apparent that this is not true. Primarily this is due to the competing noise sources, laser phase noise and accelerometer noise which are both significantly larger than quantum noise. This means that techniques which typically aid phase estimation through the reduction of quantum noise won't help satellite geodesy in its current form. However, at some point in the future such missions may be quantum noise limited. We now numerically investigate this regime with a full 3D model to support the results from our 1D model presented in the main text.

A key difference between a quintessential phase estimation problem and satellite geodesy is that in phase estimation we typically wish to estimate a single number, which is easily extracted from the measurement results. However, in satellite geodesy the quantity of interest is much more complex. The Earth's gravitational potential is normally written as an expansion of the spherical harmonics.

$$V(r,\theta,\phi,t) = \frac{\mu}{r} + \frac{\mu}{r}\sum_{l=2}^{\mathrm{N_{max}}}\left(\frac{a_e}{r}\right)^l \bar{P}_{lm}(\sin(\theta)) \times [\bar{C}_{lm}(t)\cos(m\phi) + \bar{S}_{lm}(t)\sin(m\phi)] \,, \tag{65}$$

where θ and ϕ are latitude and longitude respectively, r is the distance from the satellite to the Earth's centre of mass, μ is the gravitational constant of the Earth, a_e is the mean equatorial radius of the Earth, $\bar{P}_{lm}(\sin(\theta))$ are the fully normalised associated Legendre polynomials of degree l and order m, and $\bar{C}_{lm}(t)$ and $\bar{S}_{lm}(t)$ are the fully normalised spherical harmonic coefficients of the Earth's gravitational potential. The time dependent spherical harmonic coefficients is what the GRACE-FO mission estimates.

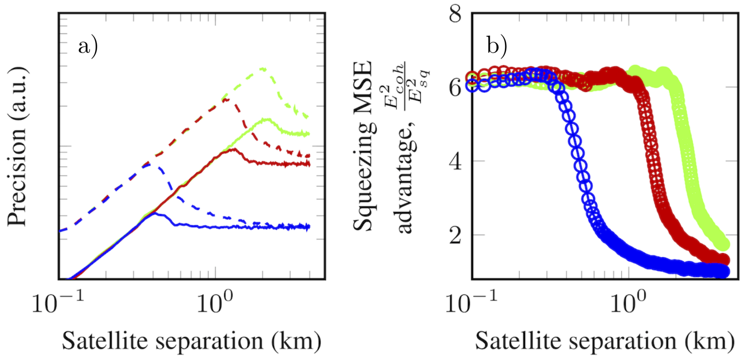

Supplementary Figure 5. **Numerically attained precision for estimating the C_{55} coefficient of the Earth's gravitational field as a function of satellite separation for different receiving aperture radii**. a) Achievable precision for several different receiving aperture radii both with and without squeezed light. b) Advantage of using squeezed light for satellite geodesy is restricted to a small region, which can be extended by increasing the receiving aperture radius. These simulations assume quantum noise is the dominant noise source. Green, red and blue lines correspond to 25 cm, 15 cm and 5 cm receiving aperture radii respectively. Dashed lines in (a) correspond to using squeezed light.

In order to numerically verify our analytic results from the 1D model we simplify the problem by estimating only a single coefficient of the Earth's gravitational field, i.e. one $\bar{C}_{lm}(t)$ or $\bar{S}_{lm}(t)$term. We consider a pair of satellites flying in a potential governed by Supplementary Equation. (65). It is assumed that we have a prior model of the Earth's gravitational field, i.e. a set of known coefficients, $\bar{C}_{lm}$ and $\bar{S}_{lm}$. The motion of a pair of satellites is simulated in this known potential. From this model we then vary one coefficient by a small amount, approximately 1% and numerically calculate the motion of the satellite pair in the new unknown potential. Quantum noise is added to the true motion of the satellites to give the measured range and based on this we perform a least squares fitting to update our model with a new estimate of $\bar{C}_{lm}$. A major simplification which we make is that we know which coefficient has changed. We define the error as $E = \left|\bar{C}_{lm} - \hat{\bar{C}}_{lm}\right|/\bar{C}_{lm}$ and precision as the inverse of the error, where $\hat{\bar{C}}_{lm}$ is the estimate of the updated coefficient. As we are assuming quantum noise is the limit, the receiving aperture size, a, plays a key role in determining the achievable precision and optimal satellite separation as shown in Supplementary Figure. 5. As predicted in the main text using our 1D model, in the quantum noise limited regime the optimal satellite separation occurs at the point where diffraction loss first becomes significant. In this regime squeezed light can offer a major advantage. The advantage from using squeezing, shown in terms of reduction in mean squared error (MSE), is assuming that mHz squeezing is available. Although a high squeezing level at mHz is currently unattainable on Earth, this may be easier to achieve in space due to the absence of seismic noise.

We next show that, as predicted by the 1D model in the main text, the way in which the quantum noise limit is reached plays a key role in determining the optimal satellite separation. Prior to now, we have assumed that the quantum noise limit is reached through instrumentation improvement such as an increase in laser stability and a reduction in accelerometer noise. However, if the quantum noise limit is reached using TDI then the optimal satellite separation is very different. Supplementary Figure. 6 shows the relative precision for estimating the C_{55} coefficient of the gravitational field as a function of satellite separation when TDI is employed. Using TDI the optimal separation is much larger than when TDI is not used and is considerably larger than the region in which squeezed light is useful. The reason for this is that TDI will strongly attenuate any gravitational signal when the satellite separations are small. Hence, the benefits of reducing the quantum noise are outweighed by the reduced signal, and the optimal strategy is a large satellite separation. TDI represents the most realistic pathway to reaching the quantum noise limit, further strengthening the argument that squeezed light may not benefit satellite geodesy for the foreseeable future.

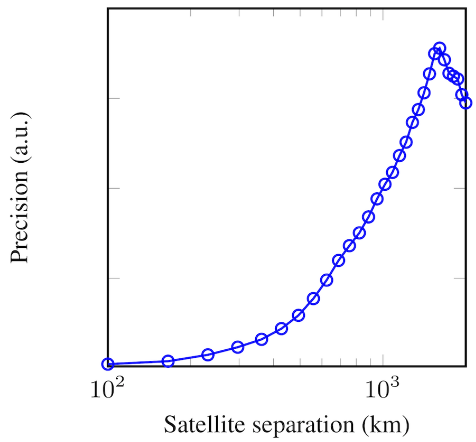

Supplementary Figure 6. **Attainable precision as a function of satellite separation when using time delay interferometry.** If the quantum noise limit is reached through TDI the optimal satellite separation is considerably larger than if this limit is reached through improvements in laser stability. The y-axis shows the relative precision in estimating the C_{55} coefficient. Reaching the quantum noise limit in this way implies that techniques from quantum optics will not aid gravitational field recovery. This figure assumes that the accelerometer noise is negligible, so that after TDI has been implemented, quantum noise is the dominant noise source.

Supplementary Note 13 . REQUIREMENTS TO REACH THE QUANTUM NOISE LIMITED REGIME

Finally, we discuss the technological improvements required before the techniques mentioned in the preceding section can be useful, i.e. what are the requirements on laser phase noise and accelerometer noise so that reducing the quantum noise is beneficial. In order to investigate this we consider laser phase noise and accelerometer noise separately, varying x_T and a_0, which characterise the two noise sources respectively.

For a given satellite orbital height and separation we compare the following two terms

$$\int_0^\infty \sqrt{S_{\text{LPN}}}|a_R(f)|df \qquad \text{or} \qquad \int_0^\infty \sqrt{S_{\text{AN}}}|a_R(f)|df \ , \tag{66}$$

and

$$\int_0^\infty \sqrt{S_{\text{QN}}}|a_R(f)|df \ . \tag{67}$$

The terms $\sqrt{S_{\text{LPN}}}$ and $\sqrt{S_{\text{AN}}}$ depend on the laser thermal noise x_T, and accelerometer instrument a_0, respectively. For the current GRACE-FO mission the terms in Supplementary Equation. (66) are considerably larger than that in Supplementary Equation. (67). However, through improvements in the laser phase noise and accelerometer noise it is possible to reach the quantum noise limit. The value of x_T or a_0 for which the terms in Supplementary Equation. (66) become equal to the term in Supplementary Equation. (67) is taken as the region when we are quantum noise limited, assuming a 5 cm receiving aperture radius, initial power, $P_0 = 25$ mW and squeezing parameter, $r = 0.8$. The above expressions look at the noise in the frequency range of interest. From Supplementary Figure. 7, we can see that up to 7 orders of magnitude improvement are needed in accelerometer instrument noise before quantum noise becomes a consideration (compared with the projected accelerometer noise for the next GRACE mission, $a_0 = 1 \times 10^{-12}\text{m s}^{-2}\sqrt{\text{Hz}}^{-1}$). The requirements on laser phase noise are less stringent. The requisite improvements for laser phase noise at small satellite separations are feasible, being approximately 3 orders of magnitude. This hints that there may be an alternative regime for satellite geodesy with small satellite separation.

Indeed, if we look at the requirements on the laser phase noise for satellites at lower orbital heights, separated by a few meters, the required laser phase noise improvement to reach the quantum noise limit becomes more attainable

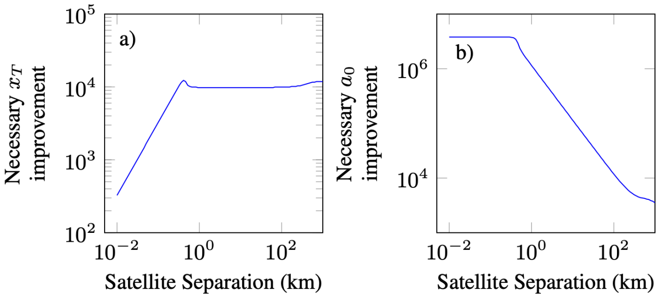

Supplementary Figure 7. **Laser phase noise and accelerometer noise improvements required to reach the quantum noise limited regime.** Assuming no squeezing, an aperture radius of 5 cm, transmitted laser power of 25 mW, $f_k = 5\times10^{-3}$ Hz for the accelerometer and a satellite orbital height of 500 km, we show the improvement in laser phase noise (a) and accelerometer noise (b) required to reach the quantum noise limited regime. We assume laser thermal noise $x_T = 1 \times 10^{-15}$ and accelerometer instrument noise $a_0 = 1 \times 10^{-12}$ m s$^{-2}\sqrt{\text{Hz}}^{-1}$ for the comparison.

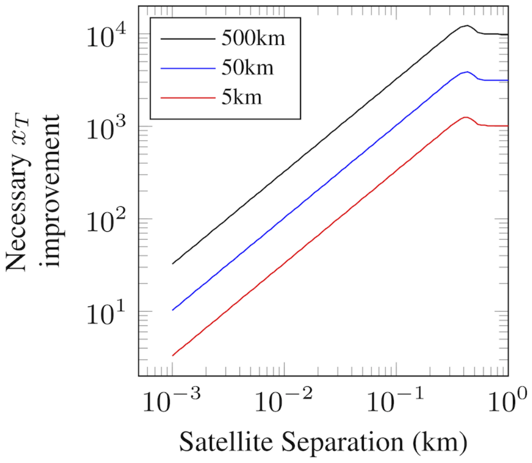

Supplementary Figure 8. **Laser phase noise improvement necessary to reach the quantum noise limit for different satellite orbital heights.** At orbital heights below 50 km the requirements to be quantum noise limited are not beyond the realms of possibility at small satellite separations. Transitioning to even lower orbital heights makes the requirement even less stringent. We again assume laser thermal noise $x_T = 1 \times 10^{-15}$ for the comparison.

as shown in Supplementary Figure. 8. The gravitational signal, Supplementary Equation. (7), falls off very rapidly at high frequencies, due to the Bessel function, $K_0(2\pi fh/v_0)$, which becomes small very quickly as $2\pi fh/v_0$ grows. This explains why transitioning to an orbital height of 50 km reduces the requirement on the laser phase noise. Lower orbital heights shift the frequency range of the gravitational signal to higher frequencies. Due to the different frequency dependence of laser phase noise and quantum noise, quantum noise is more significant at higher frequencies. Thus, a mission with a sufficiently low orbital height may be quantum noise limited. Such mission parameters are impossible for mapping the Earth's gravitational field, as the drag experienced 50 km above the Earth would be huge. However, for mapping the gravitational field of other astronomical bodies with less atmosphere, such as the Moon, or small planets, like Pluto, this becomes feasible. For example, surface pressure on Mercury is approximately 1×10^{-14} atm and on the Moon surface pressure is effectively negligible, at around 3×10^{-15} atm. As the drag force at a given height is linearly proportional to the air pressure, the drag force on these smaller bodies will be much less than that on Earth.

For mapping the gravitational field of smaller astronomical bodies, we can imagine a mission consisting of a single

long satellite with two test masses on board flying at a low orbital height. Both test masses could be placed into free-fall inside vacuum within the satellite and the distance between the two masses is measured with a laser interferometer. This is similar to the set-up used on-board LISA pathfinder [11]. In this case the need for an accelerometer is removed as both masses are in free-fall, leaving only laser phase noise and quantum noise. In this regime the laser phase noise improvement necessary before the quantum noise limit is as small as a factor of 10. Such a mission brings satellite geodesy into the realm where squeezing may be useful.

Supplementary References

[1] Spero, R. Point-mass sensitivity of gravimetric satellites. *Adv. Space Res.* **67**, 1656–1664 (2021).

[2] Abich, K. *et al.* In-orbit performance of the grace follow-on laser ranging interferometer. *Phys. Rev. Lett.* **123**, 031101 (2019).

[3] Khwaja, T. & Reza, S. Low-cost gaussian beam profiling with circular irises and apertures. *Appl. Opt.* **58**, 1048–1056 (2019).

[4] Kim, J. & Tapley, B. Simulation of dual one-way ranging measurements. *J. Spacecr. Rockets* **40**, 419–425 (2003).

[5] Armstrong, J., Estabrook, F. & Tinto, M. Time-delay interferometry for space-based gravitational wave searches. *Astrophys. J.* **527**, 814 (1999).

[6] Tinto, M. & Dhurandhar, S. Time-delay interferometry. *Living Rev. Relativ.* **17**, 6 (2014).

[7] Kroes, R., Montenbruck, O., Bertiger, W. & Visser, P. Precise grace baseline determination using gps. *GPS Solut.* **9**, 21–31 (2005).

[8] Wu, S. & Bar-Sever, Y. Real-time sub-cm differential orbit determination of two low-earth orbiters with gps bias fixing. *JPL Technical Reports Server* (2006).

[9] Flanagan, E. & Hughes, S. Measuring gravitational waves from binary black hole coalescences. i. signal to noise for inspiral, merger, and ringdown. *Phys. Rev. D* **57**, 4535 (1998).

[10] Dorner, U. *et al.* Optimal quantum phase estimation. *Phys. Rev. Lett.* **102**, 040403 (2009).

[11] Armano, M. *et al.* Sub-femto-g free fall for space-based gravitational wave observatories: Lisa pathfinder results. *Phys. Rev. Lett.* **116**, 231101 (2016).
